# Synthesis and Comprehensive in Vivo Activity Profiling of Olean-12-en-28-ol, 3β-Pentacosanoate in Experimental Autoimmune Encephalomyelitis: A Natural Remyelinating and Anti-Inflammatory Agent

**DOI:** 10.1021/acs.jnatprod.2c00798

**Published:** 2023-01-04

**Authors:** Halil Senol, Ozden Ozgun-Acar, Aydan Dağ, Ahmet Eken, Hüseyin Guner, Zaliha Gamze Aykut, Gulacti Topcu, Alaattin Sen

**Affiliations:** † Department of Pharmaceutical Chemistry, Faculty of Pharmacy, Bezmialem Vakif University, 34093 Fatih, Istanbul, Turkey; ‡ Seed Breeding & Genetics Application Research Center, Pamukkale University, 20070 Denizli, Turkey; § Department of Basic Medical Sciences, Faculty of Medicine, Medical Biology Erciyes University, 38039 Kayseri, Turkey; ∇ Department of Molecular Biology and Genetics, Faculty of Life and Natural Sciences, University of Abdullah Gul 38080 Kayseri, Turkey; △ Laboratory Animals Facility, Bilkent University, 06800 Ankara, Turkey; & Department of Pharmacognosy & Phytochemistry, Faculty of Pharmacy, Bezmialem Vakif University, 34093 Fatih, Istanbul, Turkey; □ Department of Biology, Faculty of Arts & Sciences, Pamukkale University, 20070 Kınıklı, Denizli, Turkey

## Abstract

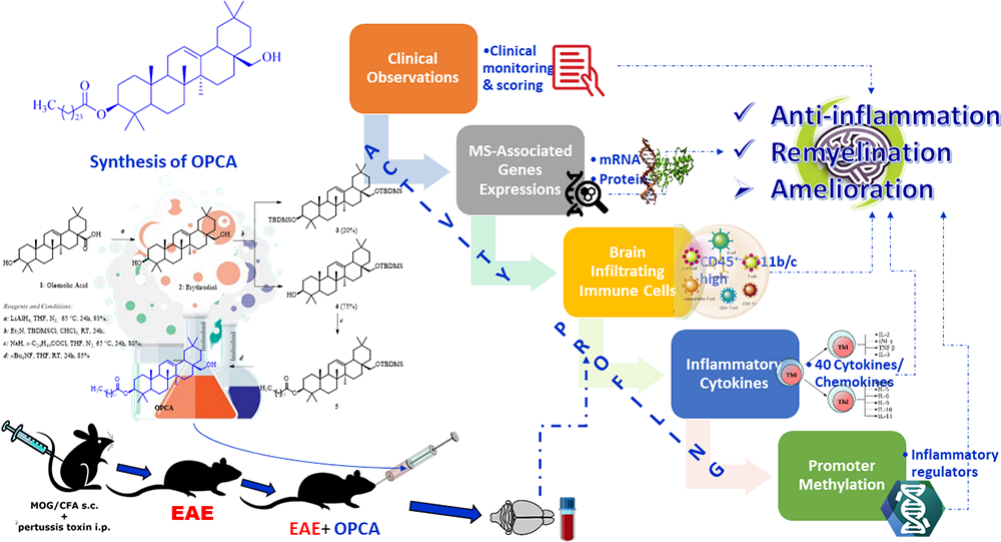

Multiple sclerosis (MS) treatment has received much attention, yet there is still no certain cure. We herein investigate the therapeutic effect of olean-12-en-28-ol, 3β-pentacosanoate (OPCA) on a preclinical model of MS. First, OPCA was synthesized semisynthetically and characterized. Then, the mice with MOG_35–55_-induced experimental autoimmune/allergic encephalomyelitis (EAE) were given OPCA along with a reference drug (FTY720). Biochemical, cellular, and molecular analyses were performed in serum and brain tissues to measure anti-inflammatory and neuroprotective responses. OPCA treatment protected EAE-induced changes in mouse brains maintaining blood–brain barrier integrity and preventing inflammation. Moreover, the protein and mRNA levels of MS-related genes such as HLD-DR1, CCL5, TNF-α, IL6, and TGFB1 were significantly reduced in OPCA-treated mouse brains. Notably, the expression of genes, including PLP, MBP, and MAG, involved in the development and structure of myelin was significantly elevated in OPCA-treated EAE. Furthermore, therapeutic OPCA effects included a substantial reduction in pro-inflammatory cytokines in the serum of treated EAE animals. Lastly, following OPCA treatment, the promoter regions for most inflammatory regulators were hypermethylated. These data support that OPCA is a valuable and appealing candidate for human MS treatment since OPCA not only normalizes the pro- and anti-inflammatory immunological bias but also stimulates remyelination in EAE.

Multiple sclerosis (MS) causes the formation of plaques characterized by local inflammation and myelin destruction in the brain and spinal cord. It is an unpredictable, chronic, and heterogeneous autoimmune disease of the central nervous system (CNS).^1,2^ As the MS advances and oligodendrocyte damage accumulates, the demyelination of neuronal axons worsens, leading to axonal loss and neuronal atrophy.^3^ MS symptoms can vary depending on the part of the brain affected by demyelination.

MS is one of the most common neurological disorders in the world. The updated MS atlas (September 11, 2020) shows that more than 2.8 million people worldwide have MS.^4^ It is the most comprehensive global survey presenting that someone is diagnosed with MS every 5 min anywhere in the world. Thus, it reveals the increase and necessity of interest in MS studies. Although the primary etiology of MS is unknown, it is accepted that a complex genetic background and environmental factors contribute together to the manifestation of the disease.^5^ It is evident that the peripheral adaptive immune cells are activated against CNS myelin epitopes.^6,7^ The paradox in the pathogenesis of MS is that the triggering event that initiates the autoimmune response is not fully understood, and there are different hypotheses on this subject.^2,8^ Experimental autoimmune/allergic encephalomyelitis (EAE) is an animal model of MS that enables experimental monitoring of the disease processes, including myelination defects, axonal pathology, and immune cell infiltration. This model has traditionally been used to imitate autoimmune demyelination in response to myelin-derived antigens like myelin oligodendrocyte glycoprotein (MOG), myelin basic protein (MBP), or proteolipid protein (PLP), functioning as an “out–in” model of the peripheral immune system activation.^9^

As a progressive CNS disease, MS does not yet have a definitive treatment, and most patients face neurological disorders after the onset of the disease.^10^ It has been shown that the drugs used today do not prevent the disease from progressing silently.^11^ Furthermore, it has resulted in an increased dependence on highly effective treatments to control both relapses and progression in the early stages of MS. Therefore, efficacious drug diagnosis studies targeting not only the immune response but also the improvement of myelination are of increasing importance.

Thus, understanding the effects of new active substances in treating MS is very important, and more studies are needed to explore them. Triterpenoids isolated from plants show potent anti-inflammatory, hepatoprotective, antioxidant, and anticancer activities.^12,13^ In this context, we isolated olean-12-en-28-ol, 3β-pentacosanoate (OPCA) from the *Capparis ovata* plant and determined in vitro effects in our previous studies.^14−16^ Herein, it was further investigated comprehensively in the EAE mouse model. First, the synthesis of OPCA in the amount that can be employed in the animal studies was performed. Then, the assessment of the molecular mechanism of action of OPCA comparatively with the positive reference drug fingolimod (FTY720) was conducted.

## Results and Discussion

### Synthesis and Characterization of OPCA

**Figure 1:**
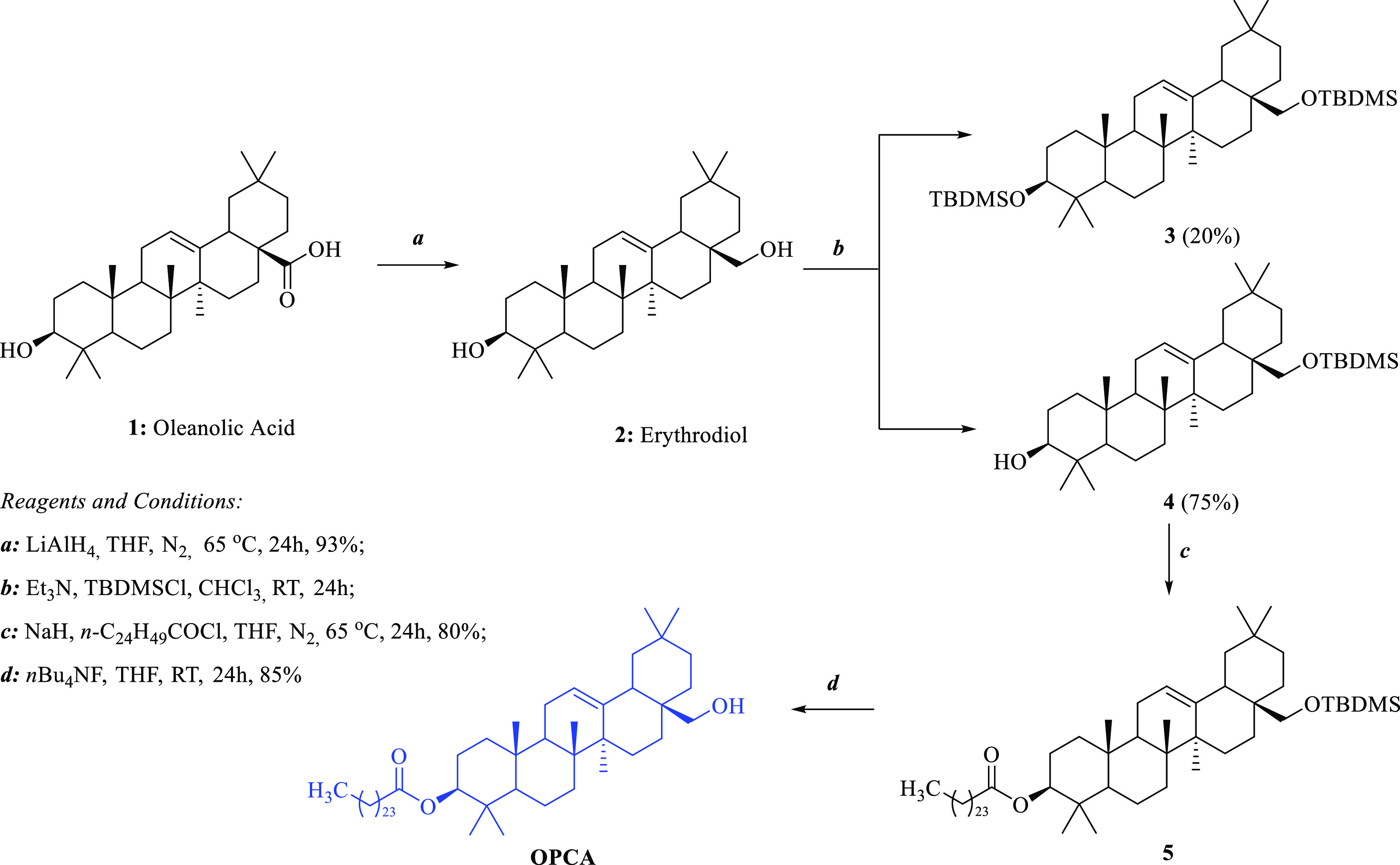
Synthesis scheme for OPCA.

Approximately 20 g of OPCA was synthesized to investigate the in vivo activity in the EAE animal model. The overall synthesis scheme is depicted in Figure 1. Purification processes of the synthesized compounds were performed by chromatographic methods. The structure of compounds was unambiguously determined by nuclear magnetic resonance (NMR) spectroscopy and high-resolution mass spectroscopy (HRMS) analyses (see Figures S1–S25 in the Supporting Information for details).

Since OPCA is an oleanane triterpene, the synthesis was started from commercially available oleanolic acid. The carboxylic acid group in the oleanolic acid was converted to primary alcohol by LiAlH_4_, and erythrodiol (**2**) was obtained. Then, the primary alcohol group was selectively protected as *tert*-butyldimethylsilyl (TMDMS) ether, and a pentacosanoyl moiety was attached to the secondary alcohol group. Finally, the removal of TMDMS by tetra-*n*-butylammonium fluoride (*n*Bu_4_NF) led to the target product OPCA in 85% yield (Figure 1, eq d). Pentacosanocyl chloride used in the reaction was prepared from its acid using SOCl_2_. During the protection of the primary alcohol in compound **2**, both alcohol groups were protected, and compound **3** occurred as a minor product. Compounds **2** and **3** were separated from each other by chromatographic methods. The NMR and HRMS spectra of the OPCA and other synthesized compounds are given in the Supporting Information. The purity of the synthesized OPCA was found to be 98.3% by HPLC analysis (Figure S26).

In the current study, first, the necessary amount of OPCA synthesis was carried out to be studied in an experimental animal model (EAE). OPCA compound was isolated from *C. ovata* by our research group and found to be anti-inflammatory in vitro.^14−16^ Oleanolic acid and derivatives have been identified in a vast array of plants in high amounts and exhibit a broad spectrum of biological and pharmacological effects.^17^ Additionally, oleanolic acid (OA) and OA derivatives are reported to be anti-inflammatory and neuroprotective and alleviate EAE-associated symptoms.^13,15,16,18^ Therefore, the present study was conducted to profile the in vivo effects of OPCA on EAE in detail.

OPCA, extraordinarily, has a very long-chain (25-C) odd-number saturated fatty acid (VLCSFA) at C-3. Little is known about the biological significance of an odd-number VLCSFA, which is very scarce due to technical difficulties inherent in their synthesis, detection, and identification. Nevertheless, their quantity reaches up to 2% in some organisms.^19^ After its initial isolation and identification in *C. ovata*, the odd-number VLCSFA group provided the impetus for further investigation.^16^ Apart from our group, there is no contribution to the literature on either OPCA or the odd-number VLCSFA addressing multiple sclerosis and autoimmunity. The results suggested that olean-12-en-3β,28-diol, 3β-pentacosanoate or 3β-pentacosanoylolean-12-en-28-ol or erythrodiol 3β-pentacosanoate [IUPAC name: (3*S*,4a*R*,6a*R*,6b*S*,8a*S*,12a*S*,14a*R*,14b*R*)-8a-(hydroxymethyl)-4,4,6a,6b,11,11,14b-heptamethyl-1,2,3,4,4a,5,6,6a,6b,7,8,8a,9,10,11,12,12a,14,14a,14b-icosahydropicen-3-yl pentacosanoate)] exerted therapeutic efficacy on the complications mentioned above.

### Stability of OPCA at Different pH

**Figure 2:**
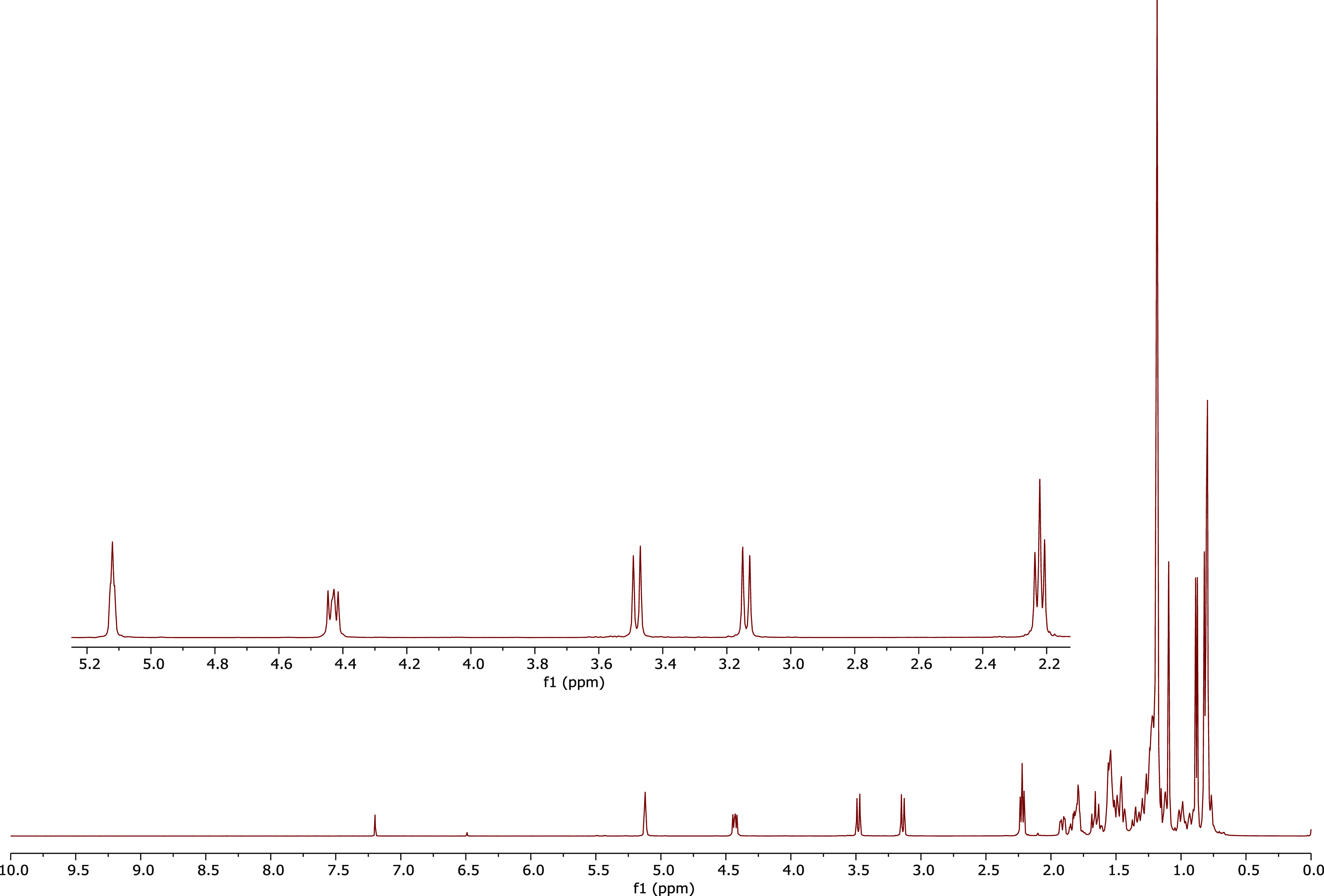
^1^H NMR spectrum of pure OPCA (500 MHz, CDCl_3_).

**Figure 3:**
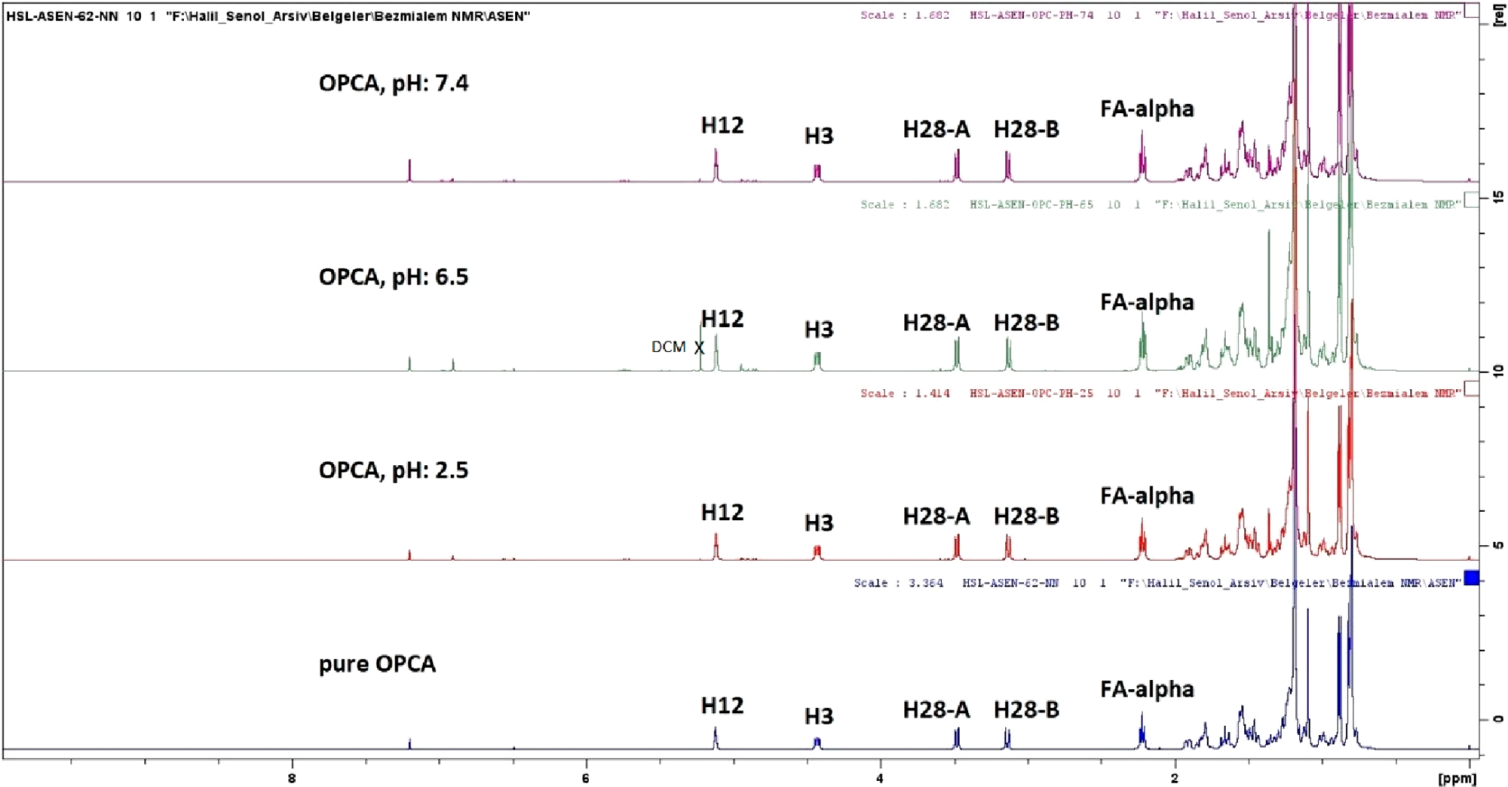
Comparison of the ^1^H NMR spectra of OPCA and treated OPCA at pH 2.5, 6.5, and 7.4 (500 MHz, CDCl_3_).

**Figure 4:**
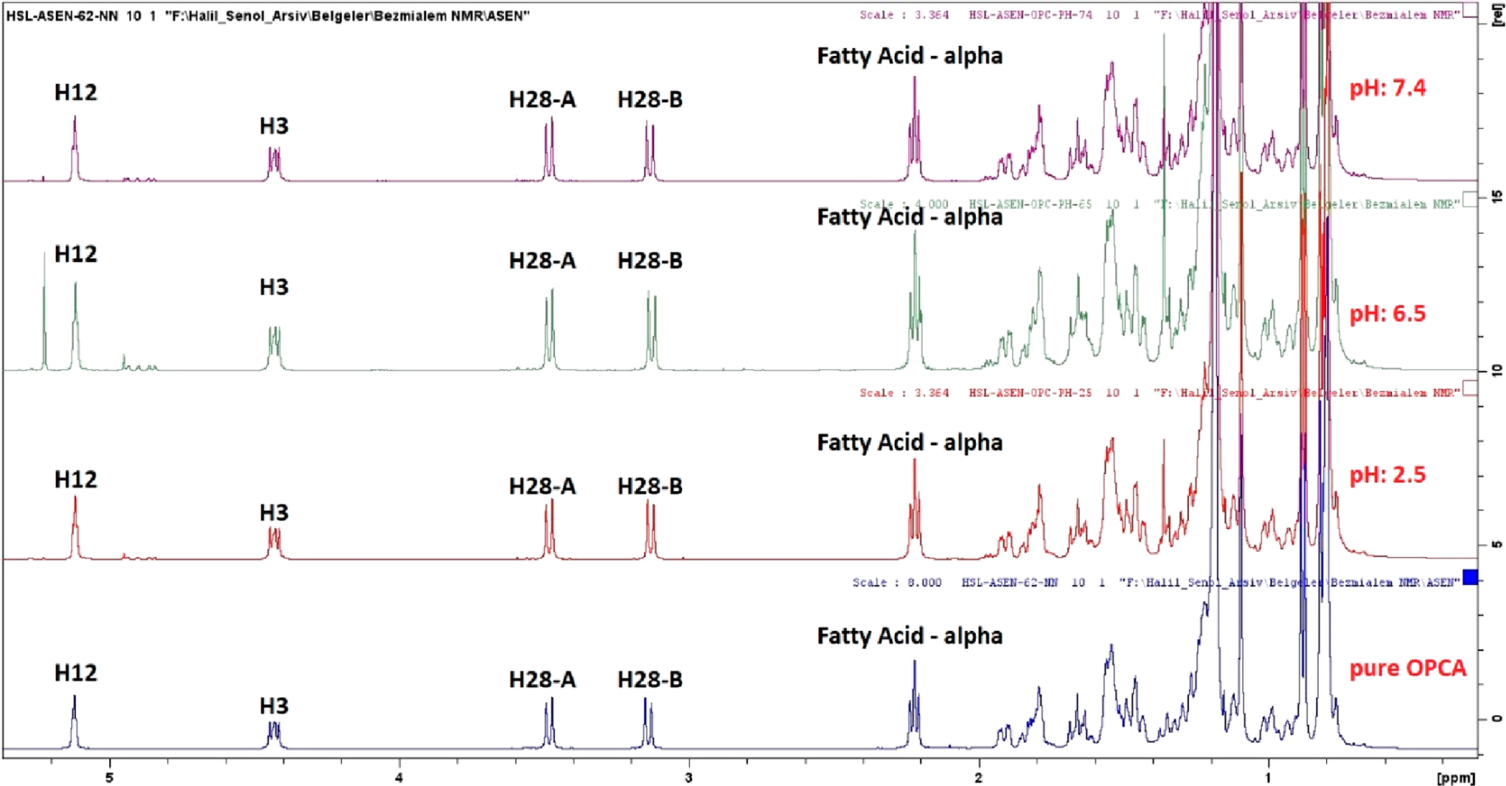
An extended comparison of the ^1^H NMR spectra of OPCA and treated OPCA at pH 2.5, 6.5, and 7.4 (500 MHz, CDCl_3_).

In order to evaluate whether or not the pentacosanoate ester moiety of OPCA is hydrolyzed in in vivo tests, OPCA was treated with buffer solutions whose pH values represented the luminal pH of the gastrointestinal system.^20^ OPCA was first dissolved in THF, added to three different phosphate buffer solutions (THF:phosphate buffer ratio was 1:50, and pH equals 2.5, 6.5, and 7.4, respectively), and stirred at 37 °C for 2 h. The mixture was subsequently diluted with water and extracted with chloroform. The organic layer was separated, dried over Na_2_SO_4_, and filtered, and the solvent was removed under reduced pressure. ^1^H NMR analysis was performed for residue and compared with the untreated OPCA. According to the NMR analysis, the ester group of OPCA did not hydrolyze at the three pH values. It was also verified from its NMR spectra that there was no byproduct of OPCA. The ^1^H NMR spectrum of untreated OPCA is given in Figure 2. The comparison of the ^1^H NMR spectra of OPCA and treated OPCA at pH 2.5, 6.5, and 7.4 is given in Figures 3 and 4, respectively.

The C-3 proton in the ^1^H NMR spectrum of OPCA resonates at 4.42 ppm. If the ester group of OPCA were hydrolyzed, erythrodiol would be produced, with a C-3 proton resonance of 3.22 ppm. It is evident that the ester group of OPCA was not hydrolyzed, as the chemical shift of the H-3 in the OPCA’s ^1^H NMR spectrum did not change. Additionally, there is no signal generation around 3.22 ppm. Consequently, it was hypothesized that OPCA was not hydrolyzed in the digestive tracts of the animals during the treatment protocols and that the observed effects were due to OPCA rather than hydrolysis products.

### Bioactivity Profiling for OPCA

#### Amelioration of EAE with OPCA: Clinical Observations

The experimental MS model (EAE) was performed twice, as stated in the Experimental Section, to investigate how the dose of OPCA will affect the immunological and inflammatory responses in the disease model. The safety dose (no observed effect level, NOEL) of 600 mg/kg bw/day for OPCA is calculated from our previous in vitro cell culture studies by applying the registry of cytotoxicity method.^21^ The dose of 0.3 mg/kg bw/day for FTY720 was taken from the literature.^22^ Then, half of the amounts were applied to the second series of animals (T2) to study the comparative effects.

To examine the therapeutic efficacy of OPCA comparatively with reference drug FTY720 on the course of EAE in mice, 600 mg/kg bw/day (T1) and 300 mg/kg bw/day (T2) OPCA and 0.3 mg/kg/day and 0.15 mg/kg/day FTY720 were fed to mice intragastrically from the 12th day of immunization, that is after observing the peak levels of clinical scores. The clinical scores were recorded blindly by two researchers every other day. However, clinical scoring could not be done every other day for the second series but every 3–4 days since, unfortunately, it coincided with the curfew due to the COVID-19 pandemic.

**Figure 5:**
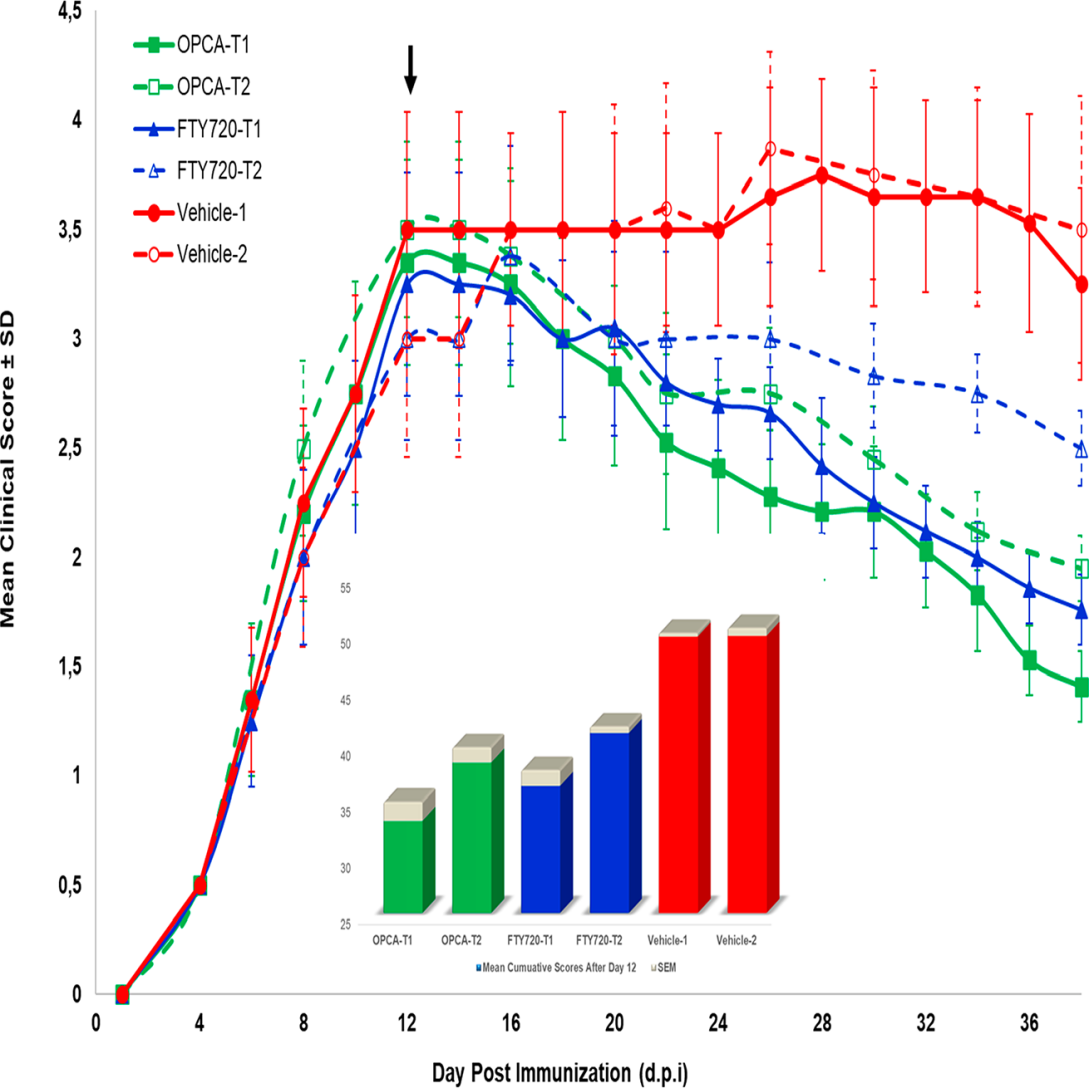
Efficacy of the OPCA in the EAE therapeutic model. Clinical scores of the EAE mice treated with vehicle, OPCA (T1: 600, T2: 300 mg/kg bw/day), and FTY720 (T1: 0.3 mg/kg bw/day, T2: 0.3 mg/kg bw/day) compound. Data are mean ± SEM (*n* = 12 (6 × 2) for the vehicle, 8 for each treatment group). The small 3D-column graph represents the cumulative clinical scores for all groups for 12–38 d.p.i.

Compared with the vehicle-treated mice, the OPCA- and FTY720-treated groups demonstrated significantly lower clinical scores starting the 14th d.p.i., while the clinical scores of the vehicle group continued to increase and stabilize (Figure 5).

The EAE-associated clinical symptoms, such as paralysis of hind limbs, were not healed in the vehicle-treated EAE mice, whereas they were significantly ameliorated in the OPCA-treated animals. Significant regenerative effects on neurological and behavioral impotence were found in the 600 mg OPCA/kg bw/day group. The cumulative scores for days 12–38 were significantly decreased in the OPCA-T1 and FTY720-T1 groups. While the lower dose of OPCA (OPCA-T2) significantly dropped the clinical scores, FTY720-T2 (0.15 mg) showed no significant differences. Similar results for FTY720 at 0.3 mg/kg bw/day were reported.^23,24^ Furthermore, the clinical scores observed with OPCA treatments at both T1 and T2 doses decreased the clinical symptoms to lower scores (around 1 with T1 and about 1.5 with T2) than with FTY720 treatments. These data showed that OPCA ameliorates the EAE progression as well as or even better than FTY720 at the applied dose regimes. The data indicate that OPCA (600 mg/kg bw/day) treatment initiated after symptom onset significantly improved neurological function better than the reference drug fingolimod (0.3 mg/kg bw/day) treatment. The following section will also address additional evidence supporting this assertion.

#### Lactate Dehydrogenase Release into Serum of OPCA-Treated Mice

First, we tested the effect of OPCA on the leakage of lactate dehydrogenase (LDH) from tissues into the blood of nonimmunized healthy C57BL/6 mice by applying the T1-treatment protocol (600 mg/kg bw/day for 14 days). LDH is used as a general indicator of tissue damage. These experiments were only carried out with the T1 treatment protocol to reduce the number of animals needlessly killed in experiments since the T2 doses were 50% less than T1. Moreover, we have not tested the reference drug FTY720 since it was studied in detail.^22,25^Table 1 depicts the average serum LDH levels detected in untreated healthy and OPCA-T1-treated mice. No significant differences were detected between the serum LDH levels of the OPCA-treated and healthy mice. LDH activity in the serum of OPCA-T2-treated mice was determined because LDH is present in a wide variety of cell types and has become an important diagnostic measure for assessing the extent of cellular and tissue damage. The data demonstrate that OPCA has no toxic effects on mice at the studied dose and acts as a restorative therapeutic, alleviating EAE without causing any other tissue degeneration or toxicities.

**Table 1:** Effects of the OPCA-T1 on the Serum LDH Activities in C57BL/6 Mice

animal	LDH activity (U/mL)
healthy	5.96 ± 1.08
OPCA-treated (600mg/kgbw/day)	5.37 ± 1.17

#### Determination of the Expression of MS-Associated Genes at the mRNA Level: RT-qPCR

In the present study, the MS-associated genes characterized in our previous studies were analyzed to determine the efficacy of the test compounds on EAE amelioration.^14^ The expression levels of genes as a marker for inflammation, myelination, cell adhesion, adaptive and humoral immunity, and apoptosis were investigated in the left hemispheres of the EAE mice treated with vehicle, OPCA, and FTY720, comparatively with healthy mice (Table 2).

**Table 2:** mRNA Expression Levels of Selected Genes (Fold Regulation) in the Brain Tissues of EAE Mice Treated with OPCA or FTY720 Compared to Healthy Animals

		treatment protocol (fold regulation)^a^				
gene	gene ID	vehicle (*n* = 12)	OPCA-T1 (*n* = 8)	OPCA-T2 (*n* = 8)	FTY720-T1 (*n* = 8)	FTY720-T2 (*n* = 8)
Inflammation/Cytokine/Chemokine						
CCL5	NM_013653	13.28	1.27	2.69	–1.06	4.46
CXCL9	NM_008599	4.29	–1.14	1.63	–1.12	2.86
CXCL10	NM_021274	7.21	1.93	3.24	2.17	5.21
HIF1A	NM_010431	2.97	1.77	1.92	1.86	2.49
NFKB	NM_008689	4.07	1.46	1.62	1.98	2.04
STAT3	NM_011486	2.31	1.32	1.54	1.87	1.96
TNF-α	NM_013693	4.23	1.52	1.76	–1.46	2.21
Myelin Structure						
MAG	NM_010758	–4.37	–1.18	1.54	–3.16	–1.85
MBP	NM_010777	–1.25	3.80	1.01	2.28	–1.10
PLP	NM_011123	–2.98	1.14	1.90	–2.32	1.54
T-Cell Activation						
CD4	NM_013488	4.12	1.55	2.55	2.12	3.06
IL6	NM_031168	5.58	–2.32	3.18	–2.28	4.34
TGFB1	NM_011577	13.0	8.29	–1.51	10.7	1.86
Innate Immunity						
C1S	NM_144938	2.98	1.98	1.63	2.12	1.91
Apoptosis						
BCL2	NM_009741	1.81	1.68	1.76	2.12	2.24
MMP9	NM_013599	5.02	2.80	1.42	4.44	2.43
Receptor/Antigen Presentation						
H2EB1 (HLA-DRB1)	NM_009741	29.1	11.4	2.83	13.9	3.85
Cell Adhesion						
APP	NM_007471	2.11	1.28	–1.56	1.72	1.77
Others						
GFAP	NM_010277	2.87	1.79	1.17	1.92	2.62
YWHAH	NM_011738	2.54	–1.78	–1.38	–1.26	1.10

a mRNA expression level (fold regulation) for each gene in EAE mice treated with vehicle, OPCA-T1 (600 mg/kg bw/day), OPCA-T2 (300 mg/kg bw/day), FTY720-T1 (0.3 mg/kg bw/day), FTY720-T2 (0.15 mg/kg bw/day) groups compared to untreated healthy animals.

**Figure 6:**
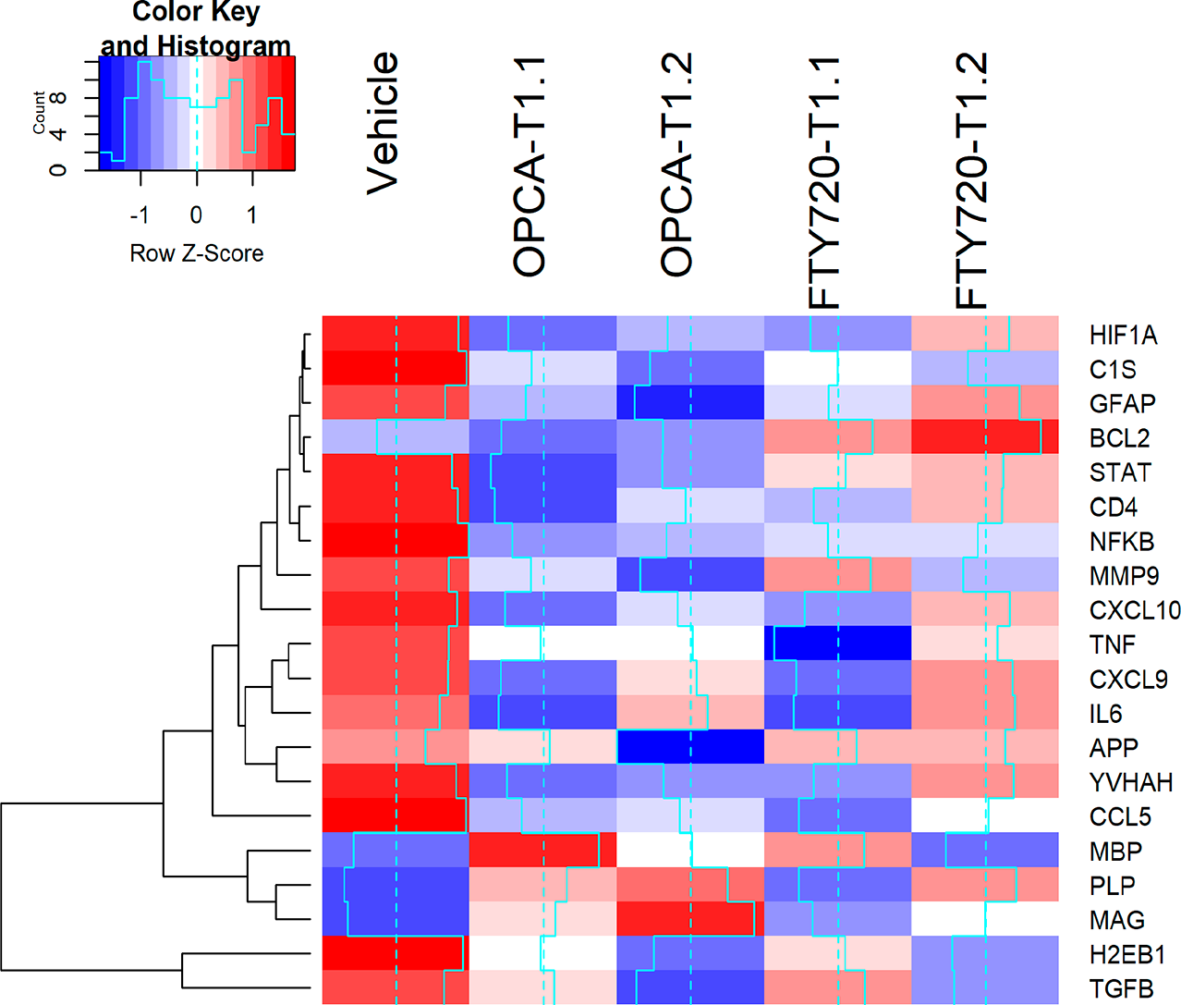
Heatmap generated from qPCR data reflecting gene expression patterns at the mRNA level for all experimental groups compared to healthy animals.

It was found that the expression level of genes associated with inflammation and the immune system was highly elevated in vehicle-treated EAE mice, while the mRNA level of genes related to myelin structure was decreased. A general gene expression pattern comparison revealed that OPCA treatments at either high or low doses significantly returned this pattern to untreated healthy mice (Table 2, Figure 6). FTY720, on the other hand, was observed to be more effective in reversing and reducing the increases observed in EAE mice, particularly in the genes associated with inflammation, such as interleukin 6 (IL6) and tumor necrosis factor alpha (TNF-α). It was determined that the mRNA level of the genes associated with myelin structure decreased (3–4-fold) in the disease model, which was not significantly altered by FYT720 treatment. Yet, OPCA showed very significant improvements in these genes (Figure 6)

More than 200 genes have been associated with MS in recent genome-wide association studies applied to large MS patient populations. A significant number of them are directly or indirectly linked to the immune system.^26^ However, it has been shown that the MOG-induced EAE models may not be optimally suited for the characterization of the function of these genes.^27^ As a result, the genes investigated in this study were chosen so that they would provide a more accurate reflection of the genetic contribution to the pathogenesis of EAE.^14^ First, it was observed that all the genes associated with inflammation and activation of the immune system, except for B cell lymphoma 2 (BCL2) and amyloid beta precursor protein (APP), were significantly increased in vehicle-treated EAE animals. On the other hand, it is seen that the expression levels of genes involved in the myelin structure are significantly reduced. Thus, it is possible to conclude that the disease model was successfully created in the subjects, as evidenced by the observed clinical scores. The increases in genes responsible for inflammation and immune activation in EAE mice were significantly suppressed by both OPCA-T1 and FTY720-T1 treatment protocols (600 mg OPCA/kg/day and 0.3 mg FTY720/kg/day, respectively). In other words, both OPCA and FTY720 showed an immunosuppressive effect. This is a common mechanism of action for FTY720 (fingolimod, or Gilenya) because it was the first oral drug approved for treating MS as an immunosuppressive and immunomodulatory agent whose immunosuppressive effects are well documented.^28^ Regarding suppressing the expression of inflammatory cytokines like CXC motif chemokine ligand 9 (CXCL9), CXCL10, and chemokine (C–C motif) ligand 5 (CCL5), FTY720 has a more considerable immunosuppressive impact than OPCA. OPCA, on the other hand, appears to be more effective than FTY720 at half doses (T2 treatment). As a result, the evidence strongly suggests that OPCA is an effective anti-inflammatory agent.

The effects of OPCA and FTY720 on the expression of myelin-related genes differ noticeably. Demyelination and associated axonal loss is one of the most common pathophysiological mechanisms in MS.^6,29^ However, whereas both OPCA treatment procedures (T1 and T2 doses) boosted the expression of key myelin-forming proteins MBP, myelin-associated glycoprotein (MAG), and PLP, no such changes were seen when using the comparator medication FTY720. These findings strongly suggest that OPCA may be a potent multitargeting agent that suppresses the immune system and remyelinates the nervous system. Currently, although some candidate drugs have been tested in MS animal models and moved to human clinical trials, there is no known remyelinating therapeutic agent in the literature.^30^ Because axonal loss and neuronal damage caused by demyelination are major causes of disability and progression in MS, OPCA could be the basis for the development of a successful therapeutic agent for improving remyelination in MS. This remyelinating effect of OPCA could explain why the EAE mice treated with OPCA had lower clinical scores and recovered faster than mice treated with FTY720.

#### Determination of the Expression of MS-Associated Genes at the Protein Level: WB Analysis

**Figure 7:**
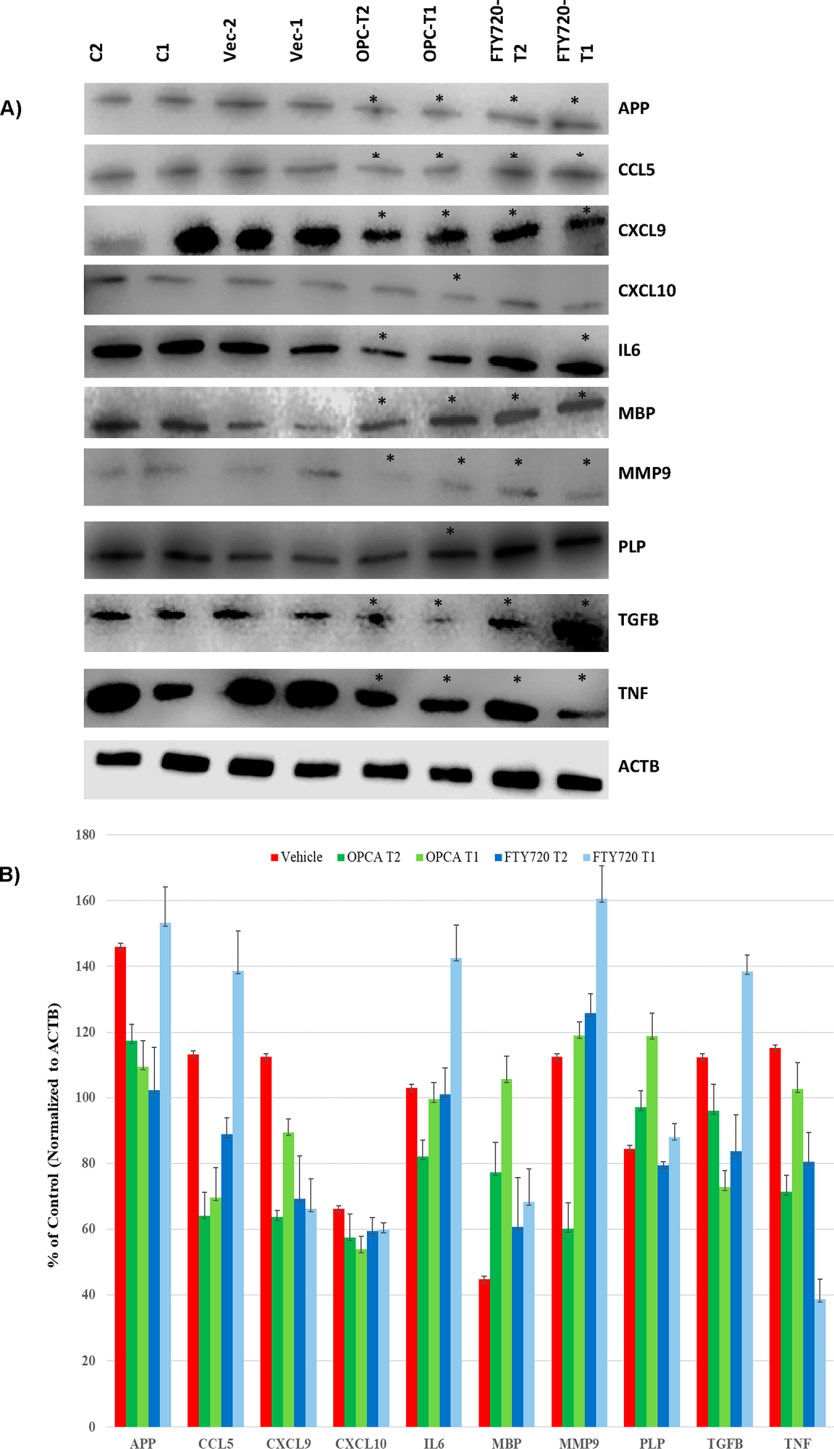
(A) Representative WB blots for the expression of APP, CCL5, CXCL9, CXCL10, IL6, MBP, MMP9, PLP, TGFB, and TNAFA in the vehicle-, OPCA-, and FTY720-treated EAE animals. The order of the samples is C2, C1, Vehicle-2, Vehicle-1, OPCA-T2, OPCA-T1, FTY720-T2, and FTY720-T. The star (*) indicates a significant difference from the vehicle-treated mice (untreated sick mice) (*P* < 0.05). For statistical analysis, the protein expression levels were measured with the densitometric scan of the blot for three independent analyses normalized with the ATCB gene [(Gene X_Treated X_/ACTB_Treated X_/(Gene X_Healthy_/ACTB_Healthy)_]. (B) Graph shows the mean of quantitative analysis for each designated bands on blots (error bars show standard deviation, *n* = 6).

The genes altered significantly at the transcript level with treatment protocols were further investigated at the protein level with WB analysis (Figure 7). Proteins extracted with the Nucleospin Triprep kit were obtained, and equal amounts of protein (100 μg) were resolved by SDS-PAGE. The separated proteins were transferred onto nitrocellulose membranes and incubated with specific primary and secondary antibodies sequentially. The blots were then developed with an enhanced chemiluminescence reagent. β-Actin (ACTB) was used to normalize total proteins.

The observed protein level variations followed mRNA levels despite a modest fold-regulation. OPCA down-regulated the protein expression levels of inflammatory genes IL6, CXCL9, CCL5, and TNF-α. The TGB1 protein level was also significantly down-regulated. The down-regulation is 2–3-fold higher than the one detected with FTY720 treatments. The expression of APP and matrix metallopeptidase 9 (MMP9) proteins affecting cell adhesion and extravasation was also down-regulated (2-fold) with OPCA treatment. In addition, the expression of myelin antigens PLP and MBP was significantly up-regulated (2-fold) with OPCA treatment, which was not affected with FTY720 treatments (Figure 7). These results indicate that OPCA not only dampens the immune activation and inflammation similar to FTY720 but also promotes remyelination, probably affecting the health of oligodendrocytes.

Studies on mRNA-protein expression levels have revealed a notoriously weak correlation between their levels of expression. As a result, only inferences drawn from mRNA expression data have received the majority of attention.^31^ In the present study, significant increases and decreases were also found at the protein level in conjunction with the data on mRNA. Despite the fact that the rise and drop levels in protein expression are not as high as in mRNA, the results show that significant correlative changes did occur. In particular, an important observation is the identification of protein profiles of myelin-related genes that confirm mRNA expression. Increases in both mRNA and protein levels of myelin-related genes, which we regard as novel for the OPCA compound, are critical in demonstrating that the compound increases myelin synthesis.

#### Determination of Brain-Infiltrating Immune Cells

**Figure 8:**
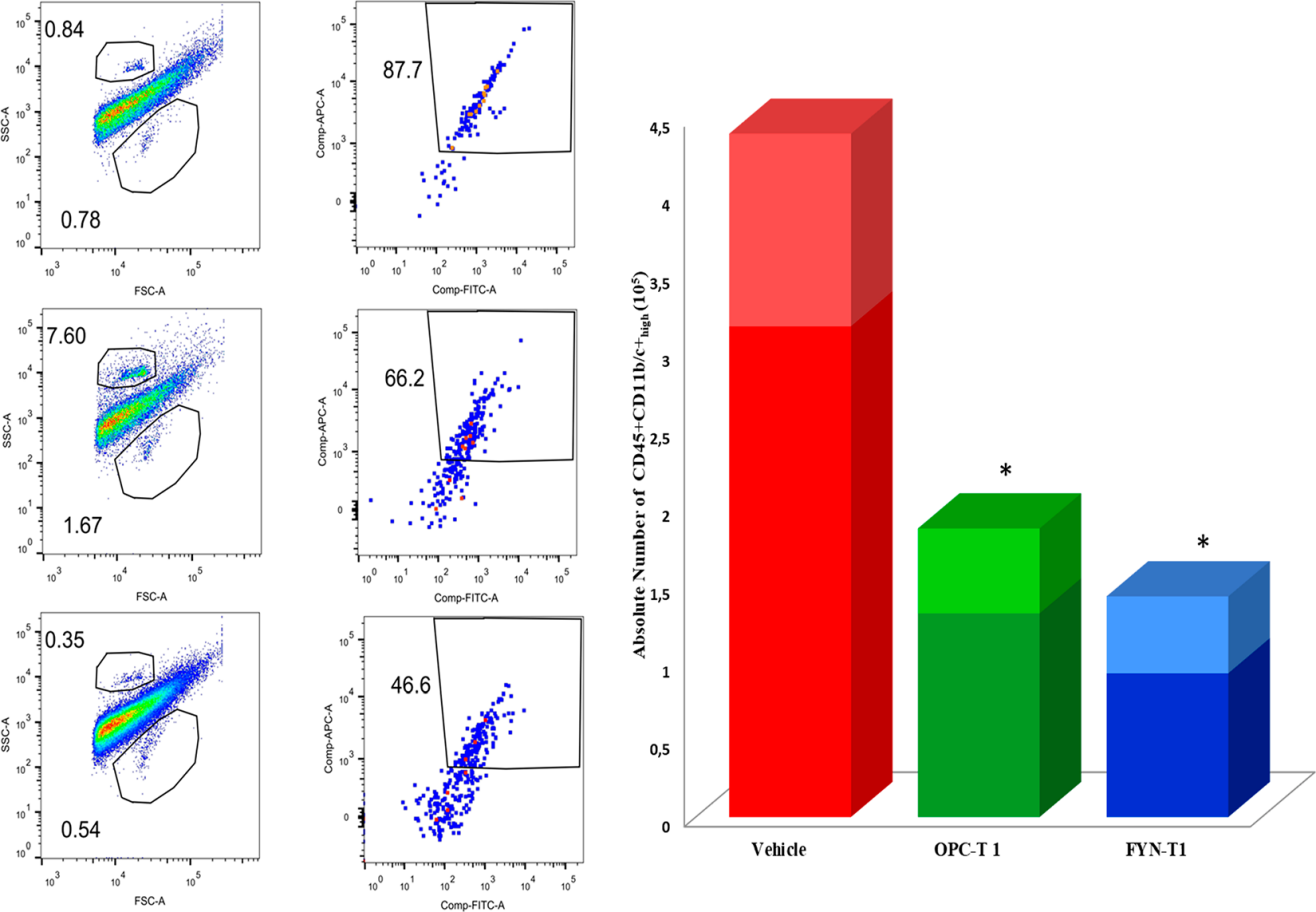
(A) Graphics displaying the gating method and group differences of CD45^+^ and CD11b/c-high cells isolated from the brain of vehicle-, OPCA-, and FTY-720-treated EAE mice. The top gate in the FSC/SSC plot shows cell counting beads, and the bottom gate shows the live leukocytes. The leukocytes in the bottom gate were further phenotyped. (B) Absolute quantitation of CD45^+^CD11b/c^+^ immune cell numbers (monocytes, macrophage, and lymphocyte cells) infiltrating the brain in the vehicle-, OPCA-, and FTY720-treated EAE mice. FSC: forward scatter, SSC: side scatter, *P < 0.05.

Flow cytometry analysis was performed to determine the density of inflammatory cells infiltrating brain tissue, as shown in Figure 8. All cells with lymphocyte common antigen (CD45) expression and appropriate side scatter (SSC) were identified as leukocytes. The CD11b/c marker identified myeloid cells, monocytes, and macrophages. Cell numbers were determined for infiltrated activated cells (CD45 positive and CD11b/c positive and high) in leukocytes (Figure 8).

In the brain of EAE vehicle-treated animals, the total number of CD45^+^CD11b/c^+^ high expressing lymphocytes, macrophages, and microglia is 4.5-fold higher than in healthy unimmunized mice. OPCA and FTY720 treatment at high dose regimes significantly reduced the number of brain infiltrating cells expressing CD45+CD11b/c-high surface markers. Only a few cells were detected in the brain of mice treated with FTY720-T1, but a higher number of cells were detected with OPCA-T1 treatment. Unfortunately, we do not have data on the brain infiltrating immune cells for the T2 protocols (second series of experiments) since it coincided with the COVID-19 curfew. Because of the travel restrictions, the fresh brain tissues could not have been transferred on the day animals were sacrificed and brought to our laboratory in Denizli from Bilkent University, Ankara, frozen in liquid nitrogen. Leukocytes were not retrieved from the frozen tissues since they were lysed in freeze–thaw processes.

The breakdown of the blood–brain barrier (BBB) and the accumulation of infiltrating peripheral cells within the CNS characterize the progression and inflammation of MS and EAE. Furthermore, identifying these cells is critical for developing therapeutic strategies that target specific immune cell populations at different stages of disease progression.^32,33^ It was reported that the expression profile of surface markers (CD45^+^CD11b/c) of CNS infiltrating lymphocytes, macrophages, and microglia represents a higher number of inflammatory cells in EAE-diseased mice than in healthy mice. The majority of inflammatory lymphocytes, macrophages, and microglia could be determined within the surface markers (CD45^+^CD11b/c) expression profile of CNS infiltrating cells in EAE-diseased mice.^34^ As a result, in the current work, CNS infiltrating cell isolation and phenotyping were carried out further to understand better the mechanisms of action of OPCA on EAE pathogenesis. Leukocyte infiltration was significantly reduced by OPCA treatment compared to FTY720. We have found a good correlation between clinical scores and the number of brain infiltrating immune cells. In addition, there was a strong correlation between the levels of MMP9 expression and infiltrating brain cells. In MS, MMP9 expression levels have been linked to demyelination because of their role in extracellular matrix degradation.^35^ Thus, the strong correlation between the number of brain infiltrating immune cells and MMP9 expression data, as well as clinical scores, strongly suggests that OPCA prevents leukocyte infiltration of the brain by preserving BBB integrity via suppressing MMP9 expression.

#### Serum Inflammatory Cytokine Profiles

**Figure 9:**
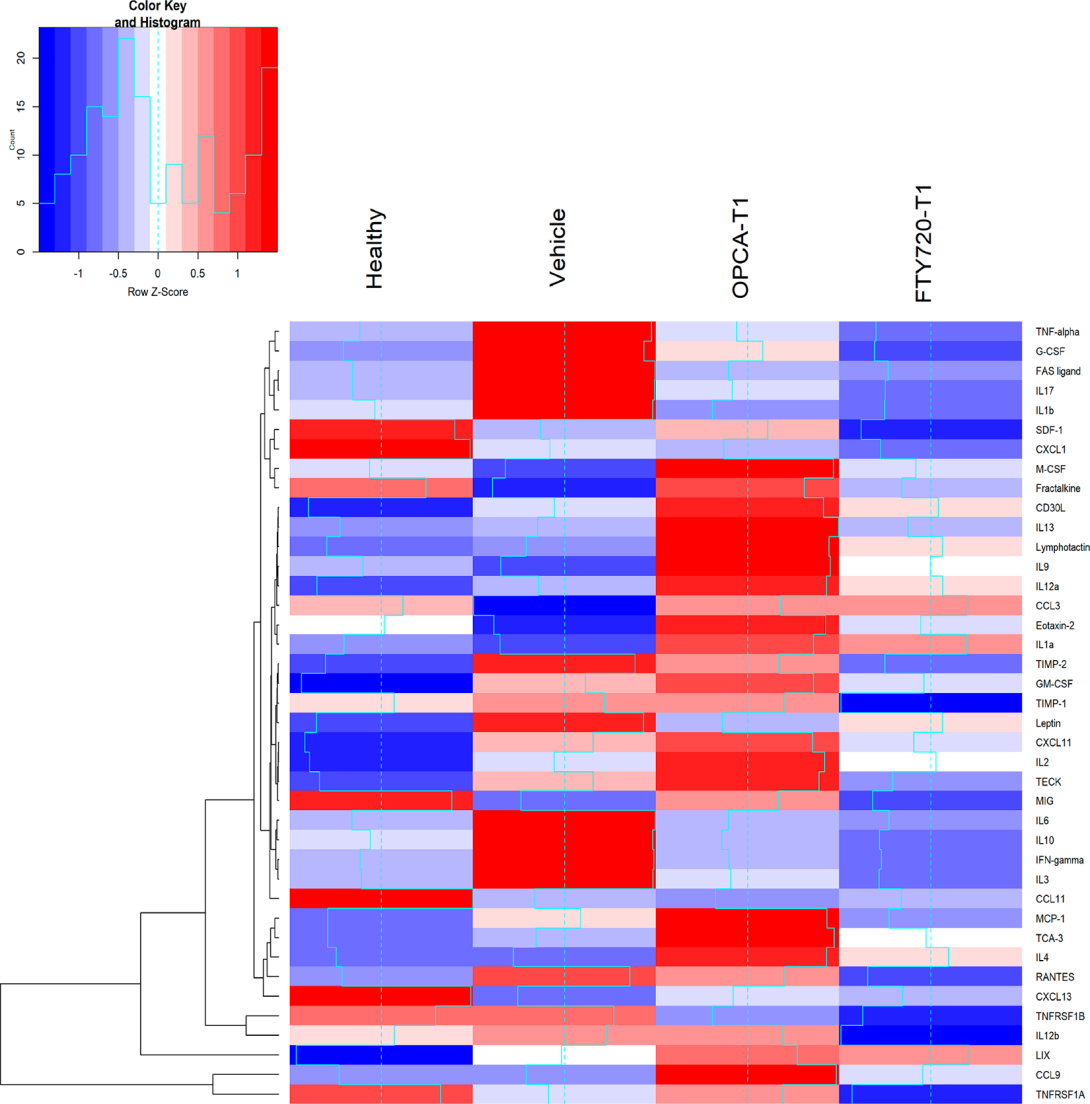
Heatmap generated from serum cytokine levels of array data obtained for each subject in the experimental groups.

To investigate the effects of OPCA on immune functions, cytokine array analyses of mouse serum were implemented and compared between vehicle-, OPCA-, and FTY720-treated mice. Data refinement was performed to obtain more accurate results, as some outliers might result from partial hydrolysis of blood cells during collections, as reported.^36^ Densitometric analysis of the abundance of each cytokine for each group is summarized as mean values in Figure 9.

**Figure 10:**
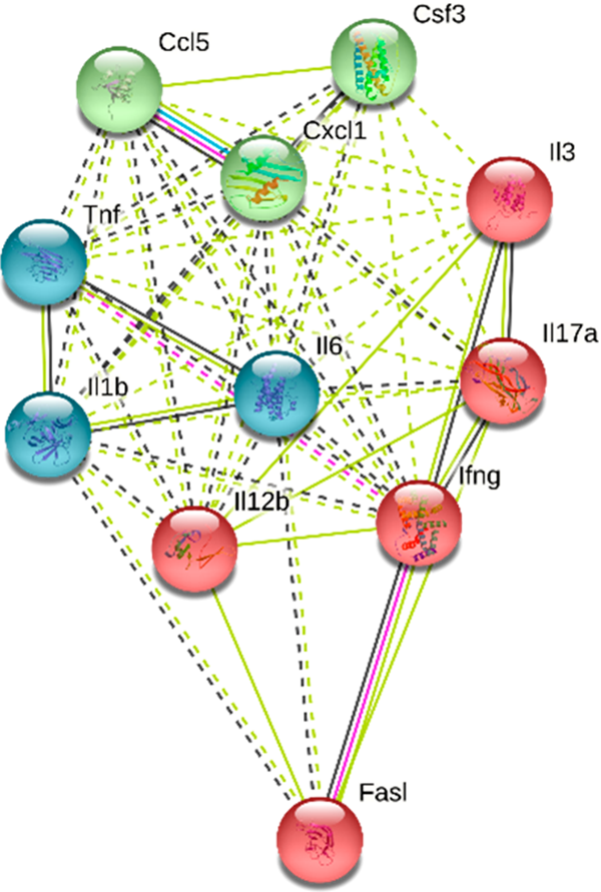
Protein–protein interaction network visualized by STRING for 11 cytokines markedly increased in EAE mice. In this view, the color saturation of the edges represents the confidence score of a functional relationship, and clustering was also done (same color cytokines primary-order interactions).

The protein–protein interaction networks of the markedly increased cytokines in EAE were created with the “string” program (Figure 10) to better understand the effect of treatments on cytokine expression.^37^ The ELISA assay for cytokines or chemokines demonstrated that serum Th1 cytokines (TNF-α, INFG, IL2), Th17 cytokines (IL17), monokines (IL1B, IL6), and peripheral chemokines (IL3, FASL, G-SCF CXCL1, CCL5) were considerably higher in vehicle-treated EAE animals, which were significantly down-regulated with the OPCA and FTY720 treatments (T1 treatment protocols).

In the present study, we discovered a marked increase in pro-inflammatory Th1/Th17 cytokine amounts of IL-17, TNF-α, INFG, and IL6 in vehicle-treated EAE disease mice (Figure 9). OPCA treatment decreased these pro-inflammatory Th1/Th17 cytokine levels slightly lower than reference drug FTY720 but statistically significant.

After demonstrating that OPCA protected BBB integrity and prevented its breakdown in EAE mice, we wondered if OPCA treatment would also prevent the Th1/Th17 cytokines/chemokines bias contributing to EAE pathogenesis. Cytokines and their receptors play an essential role in the progression of MS. The levels of pro- and anti-inflammatory cytokines have been found to correlate with changes in MS disease activity.^38,39^ Therefore, serum from mice treated with either vehicle, OPCA, or FTY720 was assessed for inflammatory cytokines. We found that both OPCA and FTY720 significantly reduced the levels of pro-inflammatory cytokines (TNF-α, IFNG, IL-6, IL17) that were up-regulated in EAE. Interestingly, in OPCA-treated mice, IL-4 and IL-10 up-regulation was observed in both sera and brain tissue compared to vehicle-treated animals. Surprisingly, the expression patterns of the cytokines TNF-α, IL-6, and CCL5 were very similar to what we found at mRNA and protein levels in brain tissues from the vehicle-, OPCA-, and FTY720-treated mice after 15 d.p.i. TNF-α is known to increase BBB permeability, and IL-1B and IL-6 activate leukocytes, all of which play essential roles in the development and progression of EAE.^39,40^ Thus, OPCA inhibits the secretion of TNF-α and other Th1/Th17 cytokines, preventing BBB permeability and leukocyte infiltration.^41^ The effects of OPCA are completely harmonious in terms of the crucial effectors that play critical roles in the EAE model.

#### Promoter Methylation Profiles of Genes Involved in the Inflammatory Responses

The promoter methylation profiles of genes involved in the inflammatory response were further studied since pro-inflammatory cytokine expression levels are known to be regulated by DNA methylation. For this purpose, bisulfite treatment was applied to the DNA isolated from mouse brains as described in the Experimental Section to detect promoter region methylation status by PCR. Genes and primer sequences are given in Supplementary Table S1. PCR products for each gene were visualized by running on a 1.5% agarose gel. The densitometric intensities of the bands were digitized using the “Scion Image” program, and the methylated/unmethylated ratios were determined (Table 3).

**Table 3:** Methylation Profile (% Mean ± SEM) of CpG Sites of the Promoter for Genes in Regulating Inflammation for Healthy and Treated Mice

genes	healthy	vehicle	OPCA-T1	FTY720-T1
CX3CL1	18.31 ± 3	13.1 ± 8	15.2 ± 3	19.0 ± 6
CXCL14	56.23 ± 6	46.5 ± 6	65.3 ± 3	73.6 ± 8
IL10RA	24.5 ± 5	34.9 ± 4	32.0 ± 7	51.0 ± 8
IL11	58.6 ± 6	61.1 ± 4	41.4 ± 4	36.2 ± 5
IL13	48.7 ± 7	57.3 ± 8	47.9 ± 3	33.1 ± 6
IL17A	55.8 ± 5	58.0 ± 6	68.3 ± 9	58.9 ± 3
IL18	25.4 ± 5	29.2 ± 8	40.5 ± 8	29.5 ± 6
IL6ST	49.95 ± 7	45.0 ± 7	44.7 ± 8	44.9 ± 7
SOCS5	6.6 ± 6	10.6 ± 4	9.3 ± 4	8.8 ± 3
TGFB1	37.9 ± 5	18.3 ± 4	39.8 ± 3	35.1 ± 6

The average percentage of methylated and unmethylated promoters in all genes pooled together reflected a complex methylation status in the experimental groups (Table 3). Promoter methylation of four genes (C-X3-C motif chemokine ligand 1 (CX3CL1), CXCL14, interleukin 6 cytokine family signal transducer (IL6ST), and transforming growth factor beta 1 (TGFB1)) was significantly reduced in vehicle-treated EAE mice. Three of these genes (CX3CL1, CXCL14, and TGFB1) were increased with the OPCA-T1 and FTY720-T1 treatment protocols while leaving ILT6ST unaltered. The OPCA and FTY720 T1 treatment protocols exerted a differential effect on the promoter methylation of interleukin-10 receptor subunit alpha (IL10RA) and IL17A. FTY720 significantly increased the promoter methylation of IL10RA, whereas OPCA did not make any significant changes compared to vehicle-treated EAE mice. FTY720, on the other hand, did not affect the IL17A promoter methylation status, whereas OPCA significantly increased it. As shown in Table 3, the OPCA and FTY720 exhibited higher methylation of pro-inflammatory gene promoters in general.

The methylation of CpG islands in the promoters of selected pro-inflammatory and anti-inflammatory genes was investigated to explore further the therapeutic effect of OPCA in the EAE model of multiple sclerosis, as studies have shown differential methylation in brain tissue collected from MS patients.^42^ Compared to untreated mice, the methylation levels of the pro-inflammatory cytokines CX3CL1, CXCL14, IL17A, IL18, and TGFB1 genes were significantly higher after OPCA administration. These increases were particularly high in the IL17A gene, even though it was lower with the control drug FTY720. In addition, changes in promoter methylation in the TGFB1 gene, for example, correlate to changes in mRNA and protein levels. Furthermore, OPCA application in the anti-inflammatory cytokine genes IL10RA, IL13, and suppressor of cytokine signaling 5 (SOCS5) caused hypo-methylation compared to untreated animals while increasing the methylation level of IL10RA with reference control drug FTY720 inexplicably. At this point, the complexity and negative consequences may be due to the heterogeneity of DNA isolated from the brain because it has been reported that the methylation profiles of neurons and non-neuronal cells in brain tissue may differ.^43^ As an epigenetic marker, DNA methylation of the promoter region GC islets was found to be altered with both OPCA and FTY720 to reduce the expression of pro-inflammatory cytokines and increase the expression of anti-inflammatory cytokines. These findings are consistent with these agents’ effects on mRNA expression and serum cytokine levels.^24^

Collectively, all these results demonstrated that OPCA, a pentacyclic triterpenoid with a very long-chain, odd-number unsaturated fatty acid, is a multitargeting therapeutic agent exerting immune suppressive and myelin regenerative effects in the CNS of a MOG_35–55_-induced EAE mouse model of multiple sclerosis. In conclusion, OPCA appears to have therapeutic potential for neurodegenerative autoimmune diseases and may contribute to developing novel therapeutics for remyelination, which is a challenging approach for demyelinating diseases such as multiple sclerosis. However, the conclusions acquired from this investigation need to be supported by further clinical studies in the future.

## Experimental Section

### Materials

Silica gel column chromatography was used in the chromatographic separations. The column chromatographies were monitored by thin-layer chromatography (TLC). Detection of spots was conducted using UV light, a 10% cerium(IV) sulfate solution in sulfuric acid, and heating in the oven at 100 °C. NMR analyses (^1^H NMR and ^13^C-APT NMR) were used to determine the chemical structures. HRMS analyses were performed for the determination of molecular weight.

^1^H NMR and ^13^CAPT NMR spectra were recorded by a Bruker Avance NEO NMR spectrometer at 500 and 125 MHz, respectively. Coupling constant values are given in hertz (Hz). Chemical shifts are reported in δ (parts per million) units relative to the internal standard tetramethylsilane (δ = 0.00 ppm), and the peak splits are described as follows: s (singlet), d (doublet), t (triplet), q (quartet), m (multiplet), bs (broad singlet), dd (doublet of doublets), and dt (doublet of triplets). HRMS spectra were recorded using a Thermo Fischer Scientific Q Exactive Hybrid Quadrupole-Orbitrap mass spectrometer using the ESI technique. HPLC chromatograms were recorded using the Waters preparative HPLC and PDA detector.

### Chemicals and Reagents

The following chemicals were purchased from Merck S.A. (an affiliate of Merck KGaA, Darmstadt, Germany): paraformaldehyde, acrylamide, anti-rabbit IgG-HRP conjugate, bovine serum albumin (BSA), bicinchoninic acid disodium salt hydrate, sodium dodecyl sulfate (SDS), Tris, ethanol, HPLC grade methanol, sodium carbonate, sodium hydrogen phosphate, sodium dihydrogen phosphate goat anti-rabbit IgG-HRP conjugate (ab97051), goat anti-mouse IgG HRP conjugate (ab97023), antiamyloid beta precursor protein (ab32136), anti-RANTES antibody (ab189841), anti-MAG antibody (ab46803), anti-MBP antibody (ab53294), anti-MMP9 antibody (ab38898), anti-myelin PLP antibody (ab28486), and anti-TNF-α antibody (ab1793) were from Abcam (Abcam PLC, Cambridge, UK). RNeasy lipid tissue mini kit, RT2 easy first strand kit, and mouse multiple sclerosis RT^2^ profiler PCR array were bought from Qiagen (CA, USA). Maxima SYBR Green qPCR master mix (2×) was purchased from Thermo-Fermentas. Hooke kit MOG_35–55_/complete Freund’s adjuvant (CFA) emulsion PTX (cat. no. EK-2110) was purchased from Hooke Laboratories, Inc. (Lawrence, MA, USA). All other chemicals and solvents were obtained from commercial sources at the highest grade of purity available.

### Animals

The Bilkent University (Ankara, Turkey) Animal House supplied healthy 6- to 8-week-old female C57BL/6 mice. They were maintained in tiny cages at the animal care facility of the University of Pamukkale (Denizli, Turkey) at an ambient temperature of 22 ± 1 °C on a 12 h light/dark cycle. During the studies, mice had unrestricted access to standard pellet food and purified water. The researchers conducted the second series of their studies at the Bilkent University Animal House. The Institutional Experimental Animal Ethics Committee authorized all experimental procedures involving the animals that were carried out following the licensed protocols with the assistance of veterinary services (PAU-HADYEK-2016/19).

### Chemistry

#### Synthesis of Erythrodiol (**2**)

According to the literature, the known compound erythrodiol was synthesized.^44^ A two-neck round-bottomed flask was charged with freshly distilled THF (1000 mL), and LiAlH_4_ (660 mmol, 25 g, 6 equiv) was added in an inert atmosphere. After stirring for 15 min, the solution of OA (50 g, 110 mmol, 1 equiv) was added drop by drop and refluxed overnight in an inert atmosphere. The reaction was monitored by TLC, and excess LiAlH_4_ was carefully destroyed with water after completion. The gel form of the white aluminum oxide complex was filtered, and THF was removed under reduced pressure. The residue was washed with water (3 × 400 mL) and extracted with chloroform (3 × 400 mL). The organic layers were combined and dried over sodium sulfate and filtered. The solvent was removed under reduced pressure. The desired products were obtained in their purest form.

#### Erythrodiol (**2**):

^**1**^H NMR (500 MHz, CDCl_3_) δ 5.18 (t, *J* = 3.55 Hz, 1H), 3.54 (d, *J* = 10.90 Hz, 1H), 3.20 (d, *J* = 10.90 Hz, 1H), 3.21 (dd, *J* = 11.03, 5.59 Hz, 1H), 0.98 (s, 3H), 0.93 (s, 3H), 0.92 (s, 3H), 0.87 (s, 3H), 0.86 (s, 3H), 0.77 (s, 3H); ^13^C NMR (125 MHz, CDCl_3_) δ 144.27, 122.40, 122.38, 79.03, 69.70, 55.22, 47.62, 46.52, 42.38, 41.77, 39.83, 38.82, 38.65, 36.97, 34.14, 33.25, 32.62, 31.08, 31.00, 28.14, 27.25, 25.99, 25.60, 23.64, 23.57, 22.05, 18.40, 16.78, 15.63, 15.56; ESI-HRMS formula: C_30_H_50_O_2_, exact mass *m*/*z* 442,38108, calculated [M + 1 – H_2_O]^+^*m*/*z* 425.37834, experimental [M + 1 – H_2_O]^+^*m*/*z* 425.37701.

### Synthesis of TBDMS Derivatives of Erythrodiol (Compounds **3** and **4**)

A round-bottomed flask was charged with chloroform (500 mL). Erythrodiol (10 g, 22.5 mmol, 1 equiv) and Et_3_N (5.75 mL, 45 mmol, 2 equiv) were added and stirred for 15 min at room temperature. Finally, TBDMSCl (3.75 g, 25 mmol, 1.1 equiv) was added and stirred overnight under an inert atmosphere. According to TLC analysis, two products were obtained. The reaction mixture was washed with aqueous HCl solution (5%, 250 mL) and extracted with chloroform (3 × 200 mL). The organic layers were combined and dried over sodium sulfate and filtered. The solvent was removed under reduced pressure, and the residue was adsorbed on silica gel. Two compounds were purified by silica gel column chromatography using an ethyl acetate–hexane mixture (1:9). While compound **3** (white solid, 20% yield) was eluted from the column first, compound **4** (white solid, 75%) was eluted later.

#### Compound **3**:

^**1**^H NMR (500 MHz, CDCl_3_) δ 5.12 (t, *J* = 3.8 Hz, 1H), 3.36 (d, *J* = 9.5 Hz, 1H), 3.15 (dd, *J* = 11.0, 4.6 Hz, 1H), 3.11 (d, *J* = 9.5 Hz, 1H), 2.06–1.96 (m, 1H), 1.12 (s, 3H), 0.89 (s, 2H), 0.89 (s, 2H), 0.87 (s, 3H), 0.85 (s, 9H), 0.85 (s, 9H), 0.84 (s, 2H), 0.83 (s, 2H), 0.72 (s, 3H), 0.00 (s, 6H), −0.04 (s, 6H); ^13^C NMR (125 MHz, CDCl_3_) δ 144.70, 122.14, 79.54, 69.21, 55.33, 47.71, 46.73, 42.06, 41.74, 39.88, 39.41, 38.68, 37.18, 33.35, 32.78, 31.21, 31.07, 28.61, 27.74, 25.98, 25.96, 25.54, 23.63, 22.51, 18.61, 18.27, 18.18, 16.76, 16.19, 15.61, −3.68, −4.82, −5.36, −5.38.

#### Compound **4**:

^**1**^H NMR (500 MHz, CDCl_3_) δ 5.16 (t, *J* = 3.7 Hz, 1H), 3.40 (d, *J* = 9.5 Hz, 1H), 3.26–3.18 (m, 1H), 3.15 (d, *J* = 9.5 Hz, 1H), 2.10–1.98 (m, 1H), 1.26 (s, 2H), 1.16 (s, 3H), 1.00 (s, 3H), 0.90 (s, 9H), 0.87 (s, 3H), 0.87 (s, 3H), 0.79 (s, 3H), 0.00 (s, 6H); ^13^C NMR (125 MHz, CDCl_3_) δ 144.63, 122.01, 78.93, 69.15, 55.19, 47.61, 46.67, 42.00, 41.68, 39.78, 38.77, 38.63, 37.11, 36.92, 34.38, 33.33, 32.64, 31.17, 31.02, 29.76, 28.15, 27.21, 25.93, 25.48, 23.60, 23.55, 22.41, 21.07, 18.37, 18.22, 16.70, 15.66, 15.55, −5.36, −5.39.

### Synthesis of Erythrodiol-TBDMS-Pentacosanoate Derivative (**5**)

A round-bottomed flask was charged with freshly distilled THF (300 mL), and NaH (1.60 g, 40 mmol, 4 equiv) was added. Compound **4** (5.50 g, 10 mmol, 1 equiv) was added and stirred for 10 min. Finally, pentacosanoyl chloride (10 mmol, 4 g, 1 equiv) was added, and the resulting mixture was stirred in an inert atmosphere at 65 °C for 24 h. After completion of the reaction, excess NaH was carefully destroyed with water. THF was removed under reduced pressure, and the residue was washed with water (2 × 200 mL) and extracted with chloroform (2 × 200 mL). Organic layers were combined and dried over sodium sulfate and filtered. The solvent was removed under reduced pressure, and the raw product was adsorbed on silica gel. Compound **5** was purified by column chromatography using an ethyl acetate–hexane mixture (1:19) (white solid, 80% yield).

#### Compound **5**:

^1^H NMR (500 MHz, CDCl_3_) δ 5.16 (t, *J* = 3.7 Hz, 1H), 4.58–4.46 (m, 1H), 3.40 (d, *J* = 9.5 Hz, 1H), 3.14 (d, *J* = 9.5 Hz, 1H), 2.30 (t, 7.49 Hz, 2H), 2.03 (dd, *J* = 13.6, 5.0 Hz, 1H), 1.29 (m, 42H), 1.16 (s, 3H), 0.96 (s, 3H), 0.94 (s, 3H), 0.89 (s, 9H), 0.88 (s, 6H), 0.87 (s, 3H), −0.00 (s, 6H); ^13^C NMR (125 MHz, CDCl_3_) δ 173.74, 144.70, 121.92, 80.57, 69.18, 55.24, 47.52, 46.62, 42.03, 41.70, 39.80, 38.26, 37.76, 37.12, 36.83, 34.89, 34.36, 33.28, 32.57, 31.95, 31.18, 31.04, 30.33, 29.73, 29.71, 29.70, 29.69, 29.66, 29.61, 29.49, 29.39, 29.28, 29.19, 28.07, 25.90, 25.88, 25.46, 25.20, 23.61, 23.57, 23.55, 22.72, 22.37, 18.24, 16.79, 16.70, 15.58, 14.15, −5.40, −5.43.

### Synthesis of OPCA

To synthesize the OPCA, the TBDMS protective group of compound **5** was hydrolyzed. A round-bottomed flask was charged with THF (100 mL), and compound **5** (4 g, 4.3 mmol, 1 equiv) was dissolved. A solution of TBAF in THF (2M, 4.3 mL, 8.6 mmol, 2 equiv) was added and stirred overnight at room temperature. After completion of the reaction, THF was removed under reduced pressure, and the residue was washed with water (2 × 200 mL) and extracted with chloroform (2 × 300 mL). Organic layers were combined and dried over sodium sulfate and filtered. The solvent was removed under reduced pressure, and OPCA was obtained as pure (white solid, 85% yield). Further purification was performed by recrystallization or precipitation via a chloroform–methanol mixture.

#### OPCA:

^1^H NMR (500 MHz, CDCl_3_) δ 5.12 (t, *J* = 3.7 Hz, 1H), 4.43 (dd, *J* = 9.7, 6.2 Hz, 1H), 3.48 (d, *J* = 10.9 Hz, 1H), 3.14 (d, *J* = 10.9 Hz, 1H), 2.22 (t, *J* = 7.5 Hz, 2H), 1.91 (dd, *J* = 13.5, 4.6 Hz, 1H), 1.19 (s, 42H), 1.09 (s, 3H), 0.89 (s, 3H), 0.87 (s, 3H), 0.82 (s, 6H), 0.80 (s, 3H); ^13^C NMR (125 MHz, CDCl_3_) δ 173.73, 144.28, 122.20, 80.54, 69.61, 55.22, 47.48, 46.43, 42.36, 41.69, 39.77, 38.24, 37.73, 36.92, 36.80, 34.85, 34.10, 33.21, 32.49, 31.95, 31.07, 30.94, 29.73, 29.71, 29.70, 29.69, 29.66, 29.61, 29.49, 29.39, 29.27, 29.18, 29.18, 28.04, 25.91, 25.53, 25.18, 23.60, 23.57, 23.52, 22.71, 21.93, 18.24, 16.78, 16.72, 15.57, 14.16; ESI-HRMS formula C_55_H_98_O_3_, exact mass *m*/*z* 806.75160, calculated [M + 1]^+^*m*/*z* 807.75942, experimental [M + 1]^+^*m*/*z* 807.76160.

### Stability of OPCA at Different pH

In order to evaluate whether or not the pentacosanoate ester moiety of OPCA is hydrolyzed in in vivo tests, OPCA was treated with buffer solutions whose pH values represented the luminal pH of the gastrointestinal system. Three different phosphate buffer solutions were prepared at the gastric pH value (average pH: 2.5), intestinal pH value (average pH: 6.5), and physiological pH value (pH: 7.4). A 100 mg amount of OPCA was dissolved in 2 mL of THF and added to 50 mL of 0.1 M KH_2_PO_4_–H_3_PO_4_ buffer solution (pH equals 2.5, 6.5, and 7.4 separately) and stirred at 37 °C for 2 h. The mixture subsequently was diluted with water and extracted with chloroform. The organic layer was separated, dried over Na_2_SO_4_, and filtered, and solvent was removed under reduced pressure. ^1^H NMR analysis was performed for the residue and compared with that of the untreated OPCA.

### Treatment of Animals

Experimental autoimmune encephalomyelitis was elicited in mice by immunizing with Hooke kit MOG_35–55_/CFA emulsion and PTX after mice had been acclimated to the environment for a week. A 0.2 mL amount of emulsion was injected subcutaneously at two different sites on each mouse. Following immunization, mice were given an intraperitoneal injection of 400 ng of pertussis toxin in 100 L of PBS on day 0 and day 2. Mice were monitored daily for the development of EAE and clinically rated on a scale ranging from 0 to 5 by two blinded observers for the disease’s symptoms [0 = no symptoms; 1 = loss of tail tonicity; 2 = hind limb weakness; 3 = ataxia or paresis of hind limbs; 4 = complete paralysis of hind limbs; 5 = moribund or dead].

On day 0, the mice were randomly assorted into the following four groups: group I (healthy), group II (EAE-vehicle), group III (EAE → OPCA-T1 or EAE → OPCA-T2), and group IV (EAE → FTY720-T1 or EAE → FTY720-T2). Healthy animals only received distilled water (Table 4). Following the immunization procedure outlined above, the immunized mice were randomly assigned to each group (groups II through IV). Group II is the disease group, and they received no further therapy other than the solvent (vehicle) used to dissolve the OPCA, which was 10% ethanol in sunflower oil. The treatment protocol known as the “peak protocol” was applied that began when clinical symptoms peaked (clinical scores were 3.0–3.5) and lasted for 14 days.

**Table 4:** Animal Groups and Treatment Protocols

group	descriptıion	animal #	treatment protocol
I	healthy	6 × 2	animals in this group were not subjected to any treatment, but water was given by gavage daily for 14 days to create stress in parallel with the experimental treatment protocols
II	eae→vehicle (disease vehicle control)	6 × 2	EAE was induced in animals as given above; animals were followed and monitored for 36 days from the beginning of the treatment; to create stress in parallel with the treatment protocols, the vehicle (10% EtOH in sunflower oil) was given daily for 14 days
IIIIa	EAE→OPCA T1 (OPCA treatment 1)	8	after EAE was established in animals in this group [approximately 12–14 days postimmunization (d.p.i.)], OPCA was administered by intragastric gavage at 600 mg/kg bw/day per animal for 14 days, and the animals were followed
IIIb	EAE→OPCA T2 (OPCA treatment 2)	8	after EAE was established in animals in this group (approximately 12–14 d.p.i.), OPCA was administered by intragastric gavage at 300 mg/kg bw/day per animal for 14 days, and the animals were followed
IVa	EAE→FTY720 T1 (FTY720 treatment 1)	8	after EAE was established in animals in this group (approximately 12–14 d.p.i.), FTY720 was administered by intragastric gavage at 0.3 mg/kg bw/day per animal for 14 days, and the animals were followed
IVb	EAE→FTY720 T2 (FTY720 treatment 2)	8	after EAE was established in animals in this group (approximately 12–14 d.p.i.), FTY720 was administered by intragastric gavage at 0.15 mg/kg bw/day per animal for 14 days, and the animals were followed

During the first series of treatments (T1), 600 mg of OPCA and 0.3 mg of FTY720 per kg of body weight per day were given intragastrically to animals in groups III and IV. Similarly, OPCA (600 mg) and FTY720 (0.3 mg) were administered intragastrically to group III and IV animals, respectively, during the second series of treatments (T2). The animals were euthanized humanely at the completion of the study, which included 16 h fasting. One milliliter of blood was taken from the ventricle slowly with a sterile needle and then perfused with 30 mL of physiological saline. Then the brains were extracted, and the left-brain hemispheres (without the cerebellum) were snap frozen in liquid nitrogen and kept at −80 °C until used. In contrast, the right brain hemispheres were used immediately to isolate infiltrating cells.

### In Vivo Activity Profiling for OPCA

#### Serum Lactate Dehydrogenase Activity Measurement

Serum lactate dehydrogenase levels were applied to determine whether treatment protocols had exerted any adverse effects on mice. LDH activities were determined colorimetrically with the BioVision LDH activity assay kit as described.^45^ Briefly, LDH activity was determined by measuring the increase in absorbency at 450 nm when LDH reduces NAD to NADH, which then interacts with a probe to produce a color. One unit of enzymatic activity is defined as the amount of enzyme that catalyzed the oxidation and reduction of 1 μmol of NADH per minute at 37 °C.

#### RNA, DNA, and Protein Isolation

Total RNA, DNA, and protein isolation from brain tissue were performed using the NucleoSpinTriPrep (Macherey-Nagel) mini kit for RNA, DNA, and protein purification. Thus, it ensured the parallel isolation of RNA, DNA, and protein from an undivided sample at a higher yield and purity.^46^ For this purpose, the left hemispheres of mice brains were ground to fine powders in the presence of liquid nitrogen using a DEPC-treated mortar and pestle. Then cells are lysed by incubation in a solution containing large amounts of chaotropic ions and applied to the Triprep column. DNA, RNA, and protein were isolated separately and sequentially according to the manufacturer’s instructions. The obtained RNA and DNA quality were visualized in agarose gel electrophoresis, and their concentrations were measured in the “NanoDrop” device. After RNA isolation, cDNA synthesis was performed immediately from the RNAs whose concentrations were calculated.

#### Determination of the Expression of MS-Related Genes at the mRNA Level: Real-Time Reverse Transcription-Quantitative Polymerase Chain Reaction (Real-Time RT-qPCR)

Transcriptional expression levels of the genes identified in our previous studies were analyzed to determine the efficacy of the treatment protocols in mice.^14^ For each sample, about 500 ng of total RNA in a final volume of 20 μL was transcribed to cDNA using the Accu-RT cDNA synthesis kit (ABM, Canada) according to the manufacturer’s instructions, removing possible DNA residues in the RNA extraction. Following cDNA synthesis, the volume was adjusted with sterile Milli-Q water to achieve a workable cDNA concentration of 20 ng/μL total RNA. In a 20 μL reaction volume, qPCR experiments were done using an Exicycler 96 real-time PCR System (Bioneer), identifying the target gene and glyceraldehyde-3-phosphate dehydrogenase (GAPDH)/BACT as an endogenous control. The primer sequences, adhesion temperatures, and cycle conditions are given elsewhere.^14^ The following genes were assessed: APP, BCL2, complement C1s (C1s), CCL5, cluster of differentiation 4 (CD4), CXCL10, CXCL9, glial fibrillary acidic protein (GFAP), H2-Eb1, hypoxia inducible factor 1 subunit alpha (HIF1A), IL6, MAG, MBP, MMP9, nuclear factor kappa B subunit (NFKB), PLP, signal transducer and activator of transcription 3 (STAT3), TGFB1, TNF-α, and YWHAH. Real-time PCR reactions were performed using the 2X SYBR Green (ABM) kit under our optimized conditions.

QIAGEN’s PCR Array Data Analysis Web Portal (version 3.5) was employed. Contamination with mouse genomic DNA was eliminated according to the manufacturer’s instructions and found to be less than 1%. Hence, no interferences were detected. Cycle threshold (Ct) values were used to calculate fold changes in mRNA abundance using the 2–^ΔΔCt^ method.^47^ β-Actin (BACT) was chosen as the best and least variable reference gene among the housekeeping genes tested. Changes in mRNA abundance for evaluated genes were compared between treated EAE mice (groups II, III, and IV) and healthy animals, whose mRNA abundance was set arbitrarily at 1.

#### Determination of the Expression of MS-Related Genes at the Protein Level: Western Blot Analysis

Protein fractions extracted from the samples described above were solubilized with RIPA buffer containing protease inhibitors. After determining the protein concentration by a bicinchoninic acid (BCA) assay, samples were subjected to sodium dodecyl-sulfate polyacrylamide gel (SDS-PAGE) and transferred to the nitrocellulose membrane. Briefly, 100 μg of protein samples was separated on 4% stacking and 12% separating polyacrylamide gels using the discontinuous buffer system of Laemmli.^48^ Proteins were transferred to a nitrocellulose membrane using the Trans-blot electrophoretic transfer cell (Hoeffer, USA) containing Tris-glycine/methanol buffer, pH 8.3, at 4 °C for 90 min at 90 V (at max 400 mA). Following the transfer, the membranes were blocked using 5% nonfat dry milk in Tris-buffered saline with Tween 20 (TBST) (20 mM Tris-HCl, pH 7.4, 400 mM NaCl, and 0.1% (v/v) Tween 20) for 60 min and incubated with monoclonal anti-mouse antibodies against PP, CCL5 (RANTES), CXCL9 (MIG), CXCL10 (IP10), GFAP, MAG, MBP, MMP9, NFKB, PLP, and TNF-α (diluted 1:500 in blocking solution) overnight at 4 °C. The membranes were then washed with TBST (3 × 5 min), incubated with the secondary antibody (HRP-conjugated anti-mouse IgG at a1:5000–10000 dilution) for 75 min, and again washed with TBST (3 × 5 min). Proteins were detected using SuperSignal West Pico chemoluminescent substrate (Pierce, Rockford, IL, USA), and bands were visualized using GelQuant Image Analysis Software in a DNR LightBIS Pro image analysis system (DNR Bio-Imaging Systems Ltd. Jerusalem, Israel). Protein bands were quantified using Scion Image Version Beta 4.0.2 software.

#### Determination of Brain Infiltrating Immune (Lymphocytes) Cells

The right hemispheres of mouse brains were minced into small pieces and homogenized manually by filtering through a 100 μm pluriStrainer into a 50 mL Falcon tube while continually flushing with cold Hanks’ balanced salt solution (HBSS) (+Ca/Mg). After centrifuging the homogenate at 360*g* for 5 min at 4 °C, the pellets were digested with HBSS (+Ca/Mg) containing 2 U/mL liberase at 37 °C for 30 min with continuous rotating. Thereafter, samples were filtered through a 70 μm pluriStrainer and incubated with HBSS (−Ca/Mg) containing 2000 U/mL DNase I (Boehringer-Mannheim, Mannheim, Germany) for 5 min at 37 °C. The digest was washed with HBSS (−Ca/Mg) solution and separated by centrifugation (350*g* for 5 min at room temperature). Dispersed cells were then used to isolate the CD45+ fraction using the pluriBead-cascade cell isolation system (pluriSelect Life Science, Leipzig, Germany) as described in ref (49) with an anti-mouse CD45 monoclonal antibody.

After surface staining for lymphocytes, as described in refs (32) and (34), cells were dissolved in 500 μL of FACS buffer and analyzed with FACSAria III (Erciyes University, Genome and Stem Cell Center). A 10 μL amount of “Spherotech microbead” (ACBP-100-10; 1 × 106/mL) was added to the cell suspension to calculate the absolute cell number. Myeloid cell number was calculated with the formula [(Percent Leukocyte X 2× 104/mL)/Percent “Microbead” × CD45^+^CD11b/c^+^ Cell Percent/100]. FCS files were analyzed with FlowJo. After excluding cell debris in the FSC-SSC graph, CD45^+^CD11b/c^+^ high cells were gated. Graphs of cell percentages and absolute numbers were drawn with GraphPad Prism. One way ANOVA and Dunnett’s multiple comparisons tests were applied for statistics. Results are given as a percentage and an absolute amount.

#### Serum Inflammatory Cytokine Profiles

Cytokine profiles of sera obtained from healthy and treated animals were examined using the mouse inflammation array membrane-based-ELISA kit (AAM-INF-1, RayBiotech), following the manufacturer’s protocols. Shortly after blocking the array membranes for 60 min, the membranes were incubated with 1 mL of 4-fold diluted serum at 4 °C overnight under gentle rotation. After washing with wash buffer, a biotin-conjugated primary antibody cocktail was added to each membrane and was incubated at 4 °C overnight with gentle rotation. After washing and adding horseradish peroxidase-conjugated streptavidin, membranes were probed with detection buffer and exposed to a chemoluminescent image analyzer system (Licor Odyssey XF, Lincoln, NE, USA). Density was expressed as the percentage of the detected value from the sample versus the background result. According to the manufacturer’s recommendation, the algorithm of normalized density spot values was determined by the following equation: [(normalized signal intensity for spot “X”) = (mean signal intensity for spot “X”) × (mean signal intensity of positive control spots)/(mean signal intensity of positive control spots on the reference array)]. The chemiluminescent data obtained were analyzed using the Microsoft Excel-based data analysis software tool provided free of charge by RayBiotech, and statistical analyses were performed using R 3.1.2 (www.r-project.org).

#### Promoter Methylation Profiles of Genes Involved in the Inflammatory Responses

Methylation-specific PCR (MSP) detection was applied to genomic DNA samples from healthy and treated mice. Approximately 250 ng of genomic DNA was modified with sodium bisulfite according to the instructions of the EZ DNA methylation-gold kit (Zymo Research). It yields highly pure DNA suitable for PCR analyses with a sufficiently high conversion (>99%) that was reported to be suitable for basically all biological applications.^50^ The methylation-sensitive high-resolution PCR approach was used to analyze the methylation status of the promoter region of the genes [CX3CL1, CXCL12 (SDF1), CXCL14, (FADD), IL10RA, interleukin 11 (IL11), IL13, interleukin 17 receptor A (IL17RA), IL18, interleukin-6 receptor subunit alpha (IL6RA), IL6ST (GP130), SOCS5, and TGFB1]. The MGMT gene was used as a positive control amplicon for bisulfite modification. Reaction conditions: first denaturation at 95 °C for 5 min, denaturation at 95 °C for 30 s, annealing at 60 °C for 1 min, extension at 72 °C for 1 min, 40 cycles, and extension at 72 °C for 5 min. The PCR amplification products were collected, electrophoresed on a 1.5% agarose gel, and observed under UV light. Primer sequences and annealing temperatures are given in Supplementary Table S1.

#### Statistical Analysis

Minitab 13 statistical software was used for the statistical analyses (Minitab, Inc., PA, USA). All results were presented as means, including the standard error of the means (SEM). The Student’s *t* test was used to compare groups, and *P* < 0.05 was chosen as the level required for statistical significance. One-way analysis of variance (ANOVA) was applied to evaluate statistical comparisons between the three groups. When F ratios were significant (*P* < 0.05), one-way ANOVA was employed to compare multiple groups’ means, followed by Tukey’s post hoc test. Data analysis and visualization of differentially expressed genes for microarray and qPCR analyses were performed using R 3.1.2. (www.r-project.org).

## Supporting Information Available

Spectra of compounds (PDF)

## Notes

Preliminary results were presented at the 44th International FEBS Congress in Krakow, Czechia, on July 6–11, 2019.
