# Supplementary material for: Synthesis and Comprehensive in Vivo Activity Profiling of Olean-12-en-28-ol, 3β-Pentacosanoate in Experimental Autoimmune Encephalomyelitis: A Natural Remyelinating and Anti-Inflammatory Agent
Source: J Nat Prod. 2023 Jan 4;86(1):103–18. doi: 10.1021/acs.jnatprod.2c00798 (PMC9887603; doi:10.1021/acs.jnatprod.2c00798)
Supplement: Supplementary file 1 — np2c00798_si_001.pdf [file np2c00798_si_001.pdf]

Synthesis and Comprehensive in vivo Activity Profiling of Olean-12-en-28-ol, 3β Pentacosanoate in Experimental Autoimmune Encephalomyelitis: A Natural Remyelinating and Anti-Inflammatory Agent

Halil Senol[†], Ozden Ozgun-Acar[‡], Aydan Dağ[†], Ahmet Eken[§], Hüseyin Guner[∇], Zaliha Gamze Aykut[^], Gulacti Topcu[&], Alaattin Sen[∇]*

CONTENTS

Page

Figure S1: ^{1}H-NMR spectrum of erythrodiol **(2)** (500 MHz, $CDCl_3$) 3

Figure S2: ^{1}H-NMR spectrum of erythrodiol **(2)** (extended aliphatic region) (500 MHz, $CDCl_3$) 4

Figure S3: ^{13}C-APT NMR spectrum of erythrodiol **(2)** (125 MHz, $CDCl_3$) 5

Figure S4: ^{13}C-APT NMR spectrum of erythrodiol **(2)** (extended aliphatic region) (125 MHz, $CDCl_3$) 6

Figure S5: ESI-HRMS Spectrum of erythrodiol **(2)** 7

Figure S6: ^{1}H-NMR spectrum of compound **3** (500 MHz, $CDCl_3$) 8

Figure S7: ^{1}H-NMR spectrum of compound **3** (extended aliphatic region) (500 MHz, $CDCl_3$) 9

Figure S8: ^{13}C-APT NMR spectrum of compound **3** (125 MHz, $CDCl_3$) 10

Figure S9: ^{13}C-APT NMR spectrum of compound **3** (extended aliphatic region) (125 MHz, $CDCl_3$)...........11

Figure S10: ^{1}H-NMR spectrum of compound **4** (500 MHz, $CDCl_3$)..12

Figure S11: ^{1}H-NMR spectrum of compound **4** (extended aliphatic region) (500 MHz, $CDCl_3$) 13

Figure S12: ^{13}C-APT NMR spectrum of compound **4** (125 MHz, $CDCl_3$)...14

Figure S13: ^{13}C-APT NMR spectrum of compound **4** (extended aliphatic region) (125 MHz, $CDCl_3$).........15

Figure S14: ^{1}H-NMR spectrum of compound **5** (500 MHz, $CDCl_3$)..16

Figure S15: ^{1}H-NMR spectrum of compound **5** (extended aliphatic region) (500 MHz, $CDCl_3$) 17

Figure S16: ^{13}C-APT NMR spectrum of compound **5** (125 MHz, $CDCl_3$)...18

Figure S17: ^{13}C-APT NMR spectrum of compound **5** (extended aliphatic region) (125 MHz, $CDCl_3$).........19

Figure S18: ^{1}H-NMR spectrum of **OPCA** (500 MHz, $CDCl_3$) 20

Figure S19: ^{1}H-NMR spectrum of OPCA (extended aliphatic region) (500 MHz, $CDCl_3$) 21

Figure S20: ^{13}C-APT NMR spectrum of **OPCA** (125 MHz, $CDCl_3$)..22

Figure S21: ^{13}C-APT NMR spectrum of **OPCA** (extended aliphatic region) (125 MHz, $CDCl_3$) 23

Figure S22: ESI-HRMS Spectrum of **OPCA**...24

Figure S23: ESI-HRMS Spectrum of **OPCA** (molecular ion peak)...25

Figure S24: ESI-HRMS Spectrum of **OPCA** (fragments)..26

Figure S25: ESI-HRMS Spectrum of **OPCA** (fragments)..27

Figure 26. HPLC analysis for the purifty of the synthesized OPCA. 28

Table S1: Primer sequences used for promoter methylation analyses of the inflammatory regulatory genes...30

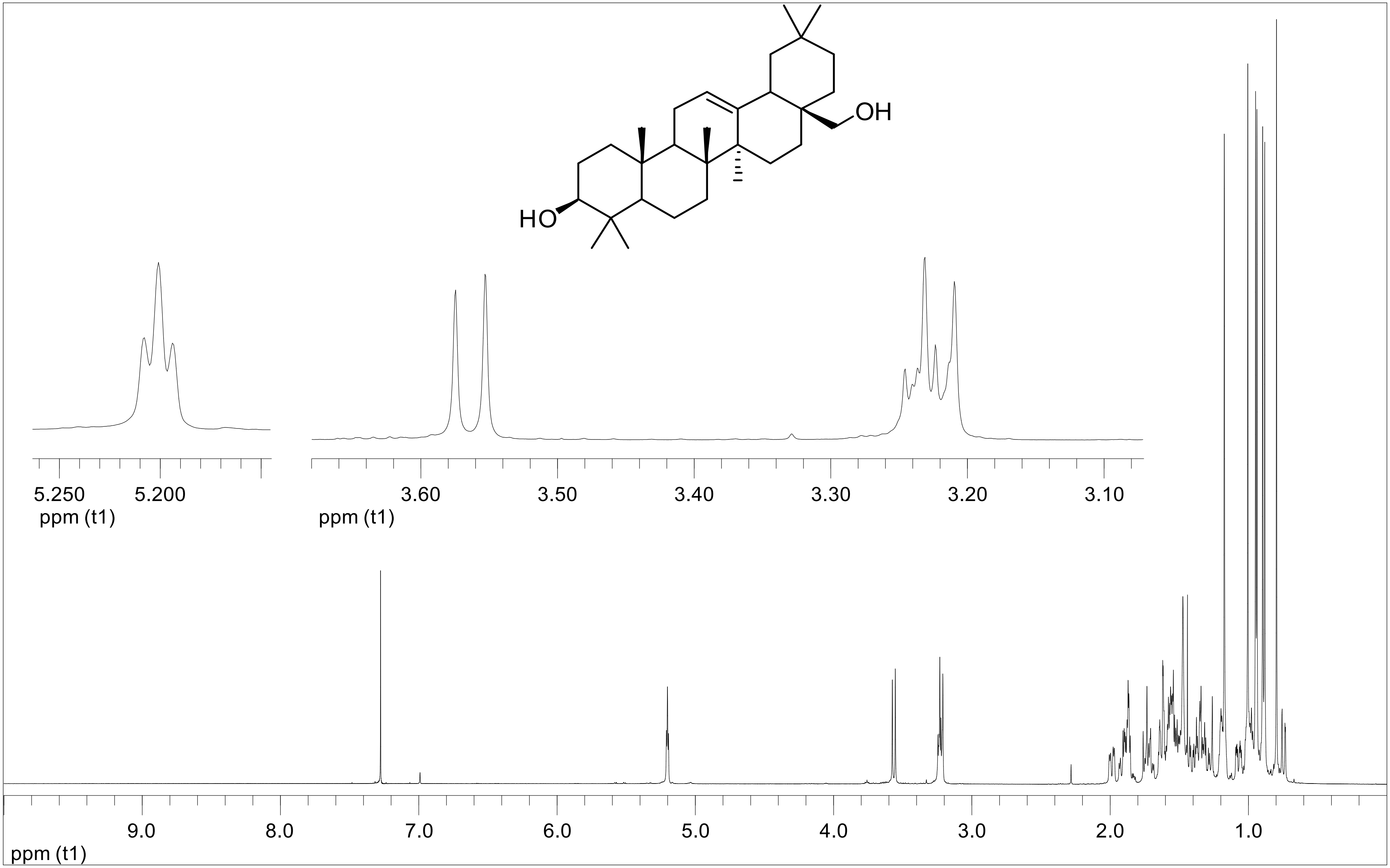

Figure S1: ^{1}H-NMR spectrum of erythrodiol **(2)** (500 MHz, $CDCl_3$)

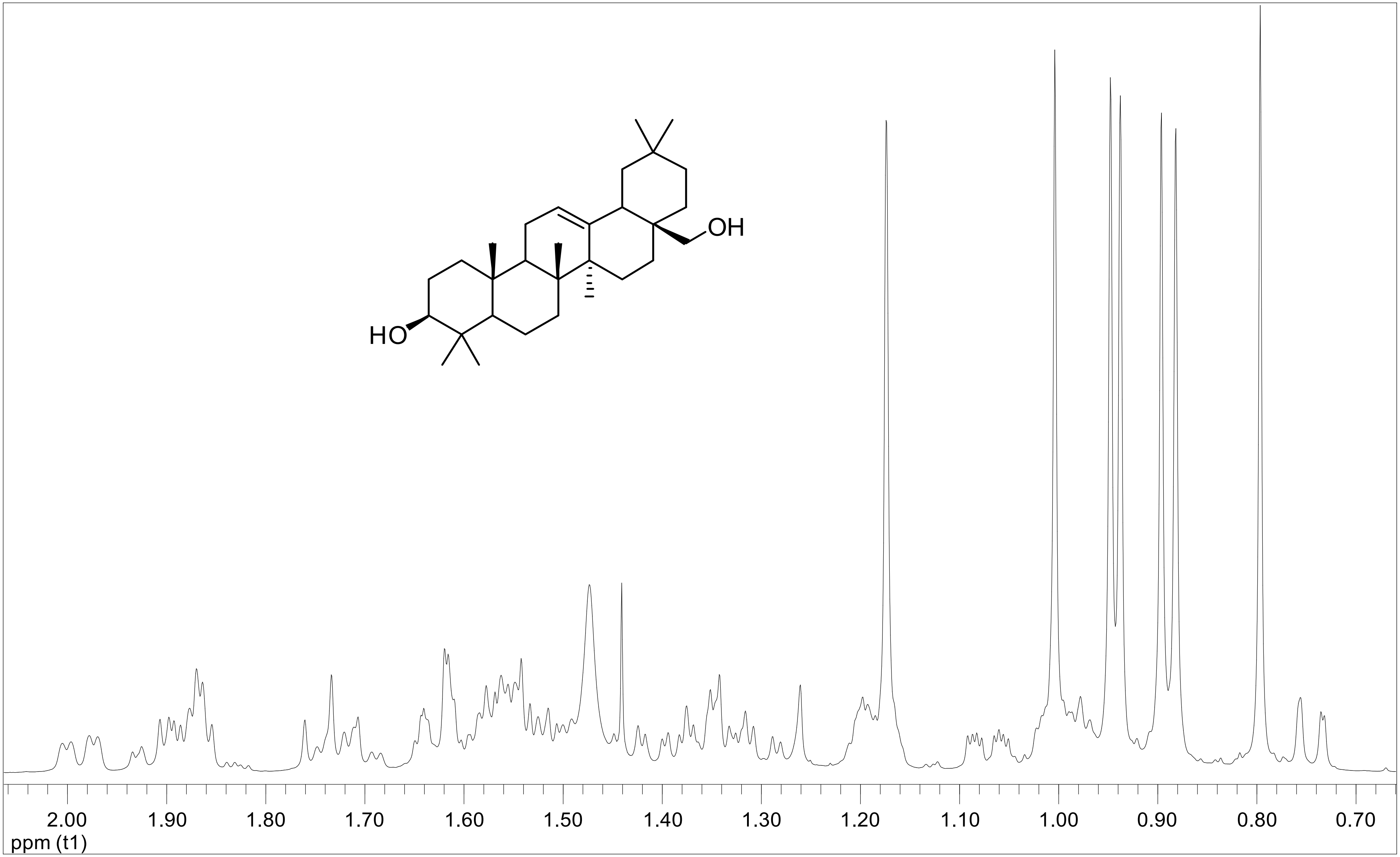

Figure S2: ^{1}H-NMR spectrum of erythrodiol **(2)** (extended aliphatic region) (500 MHz, $CDCl_3$)

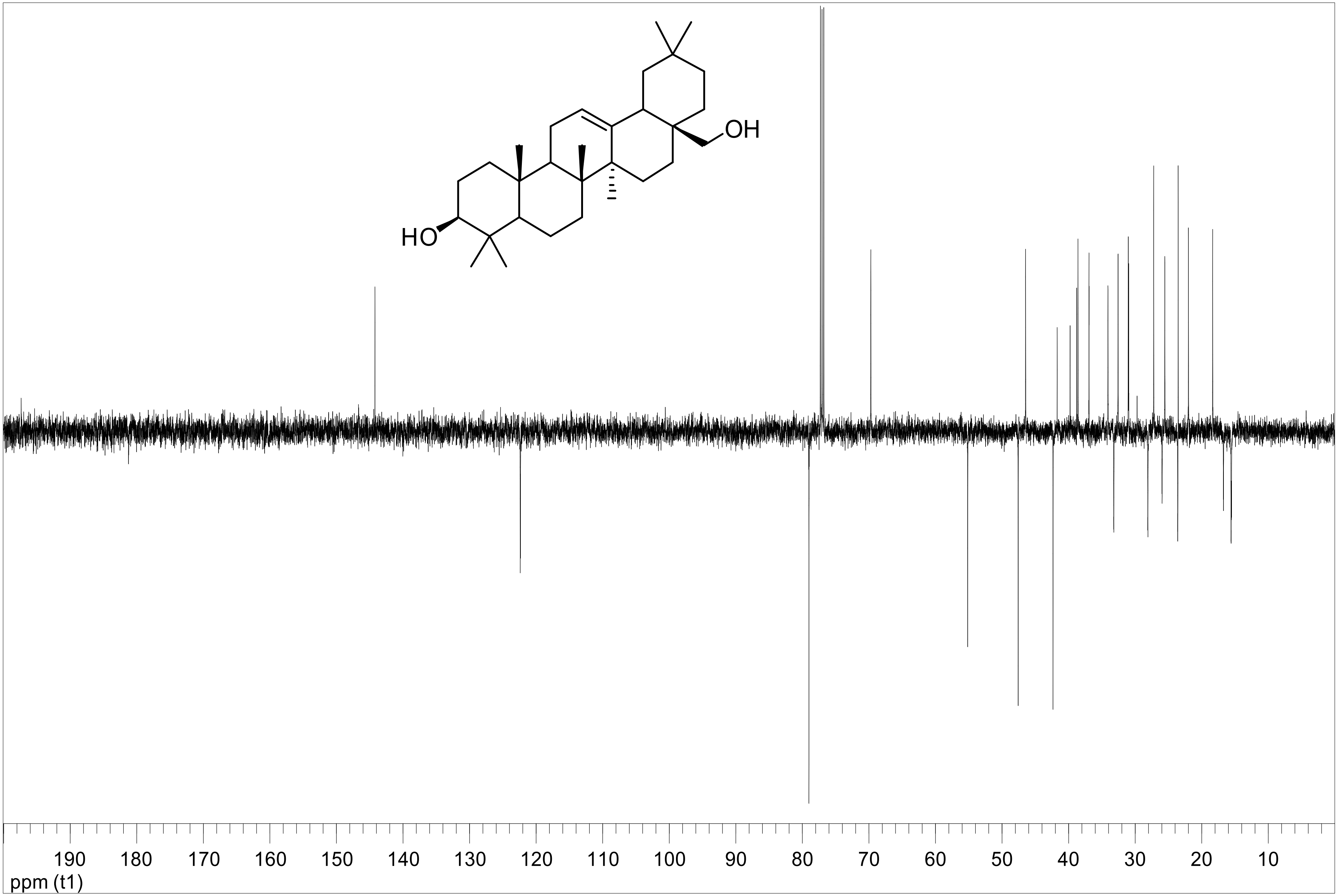

Figure S3: ^{13}C-APT NMR spectrum of erythrodiol **(2)** (125 MHz, $CDCl_3$)

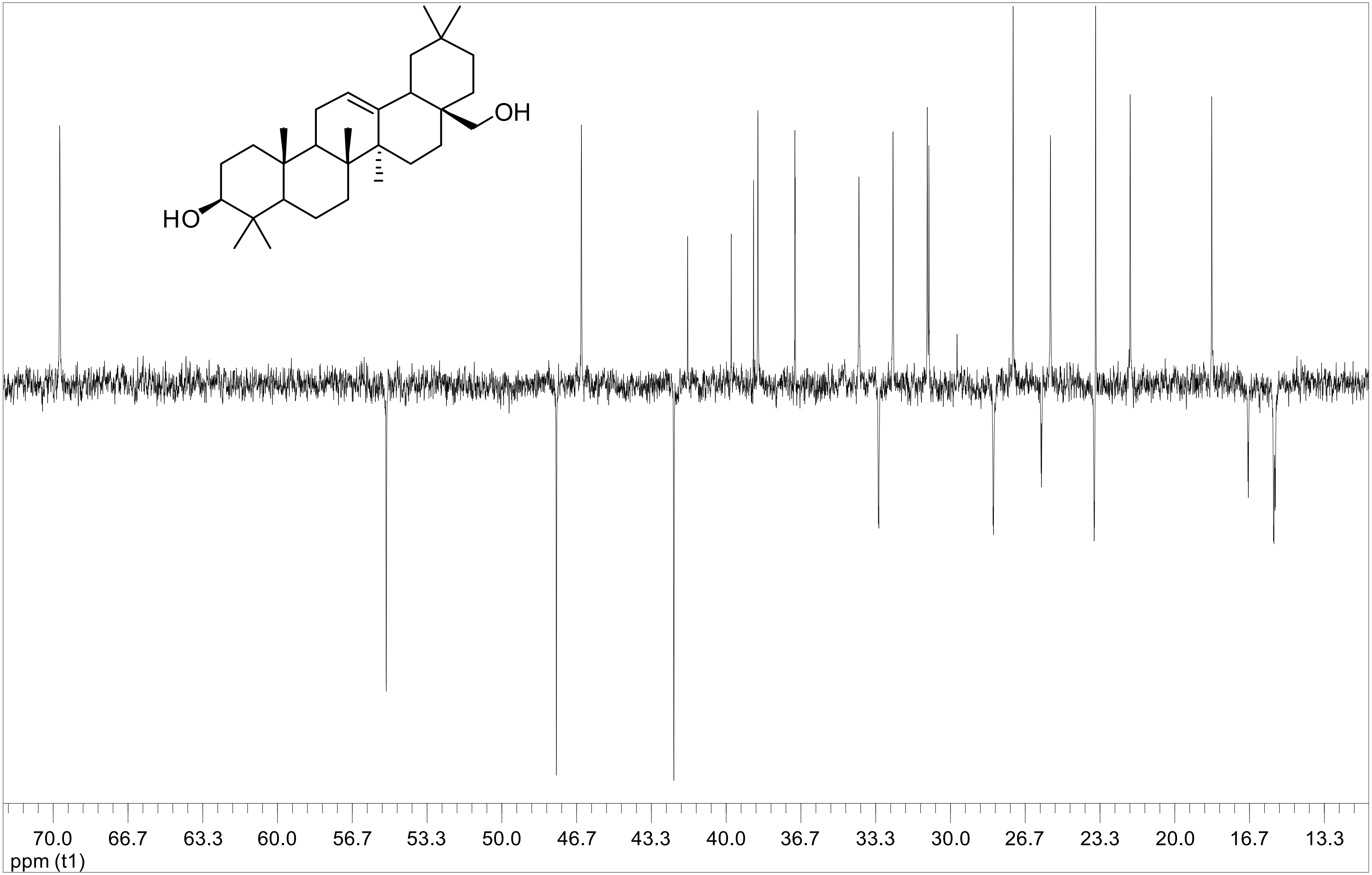

Figure S4: ^{13}C-APT NMR spectrum of erythrodiol **(2)** (extended aliphatic region) (125 MHz, $CDCl_3$)

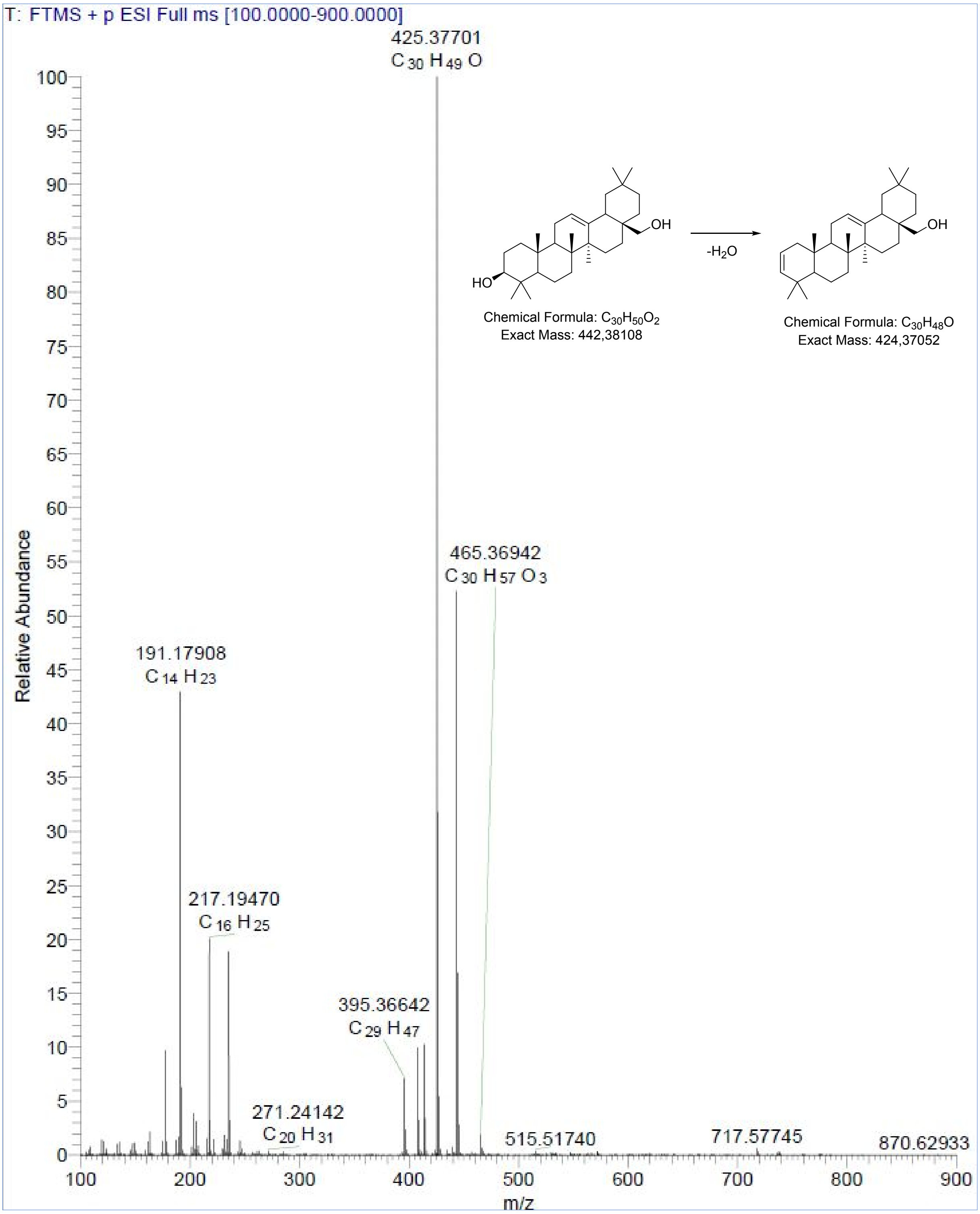

Figure S5: ESI-HRMS Spectrum of erythrodiol **(2)**

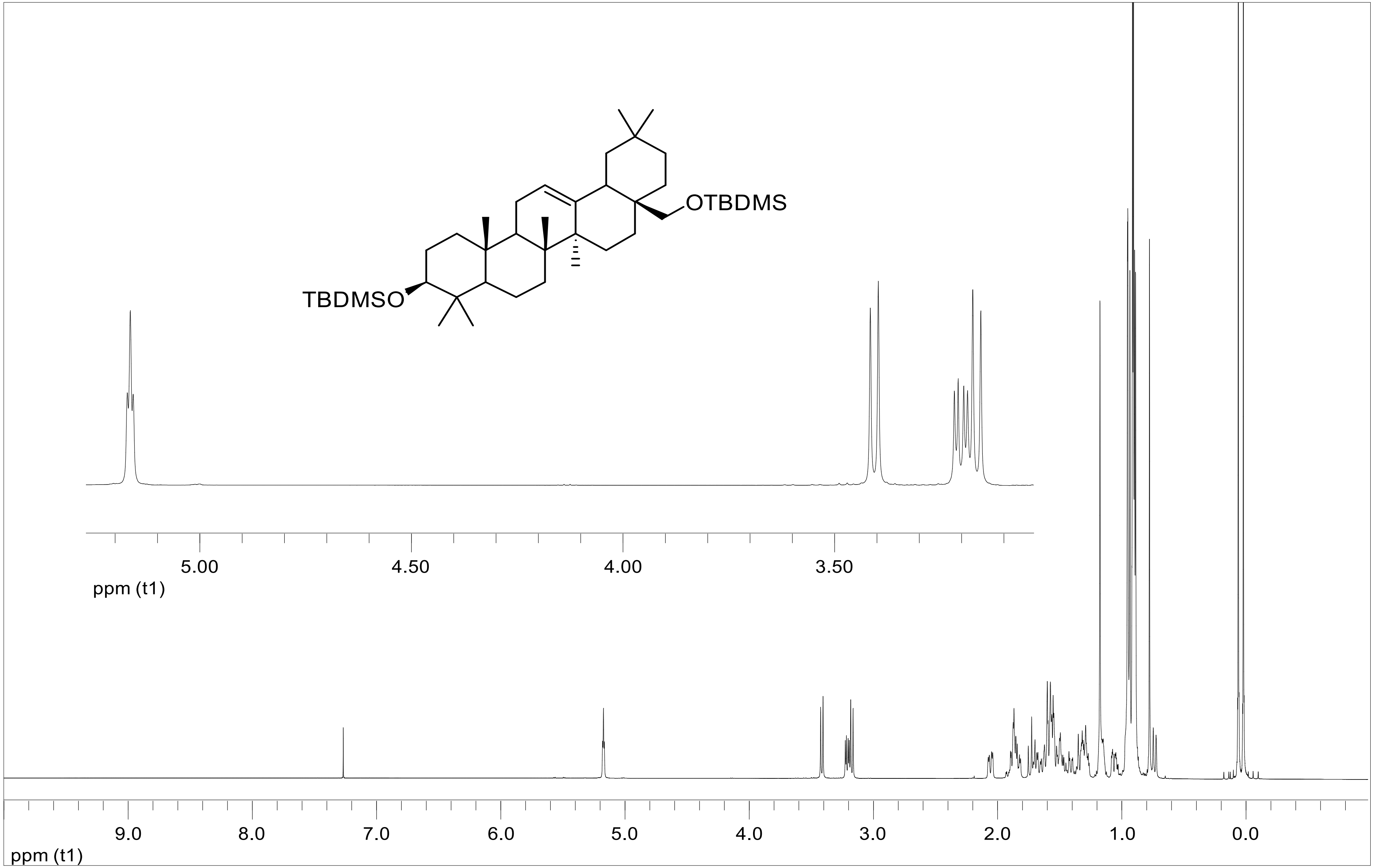

Figure S6: ^{1}H-NMR spectrum of compound **3** (500 MHz, $CDCl_3$)

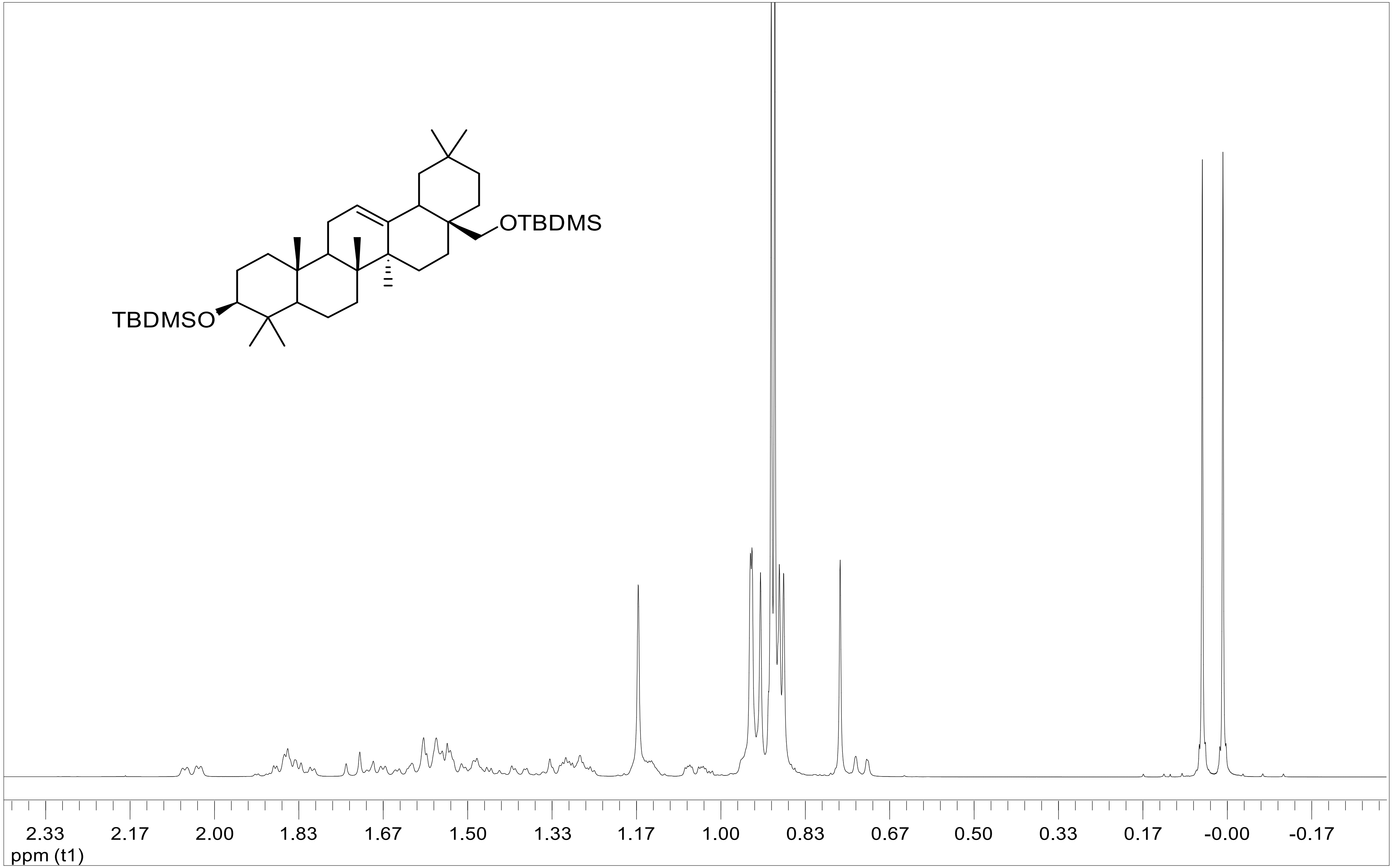

Figure S7: ^{1}H-NMR spectrum of compound **3** (extended aliphatic region) (500 MHz, $CDCl_3$)

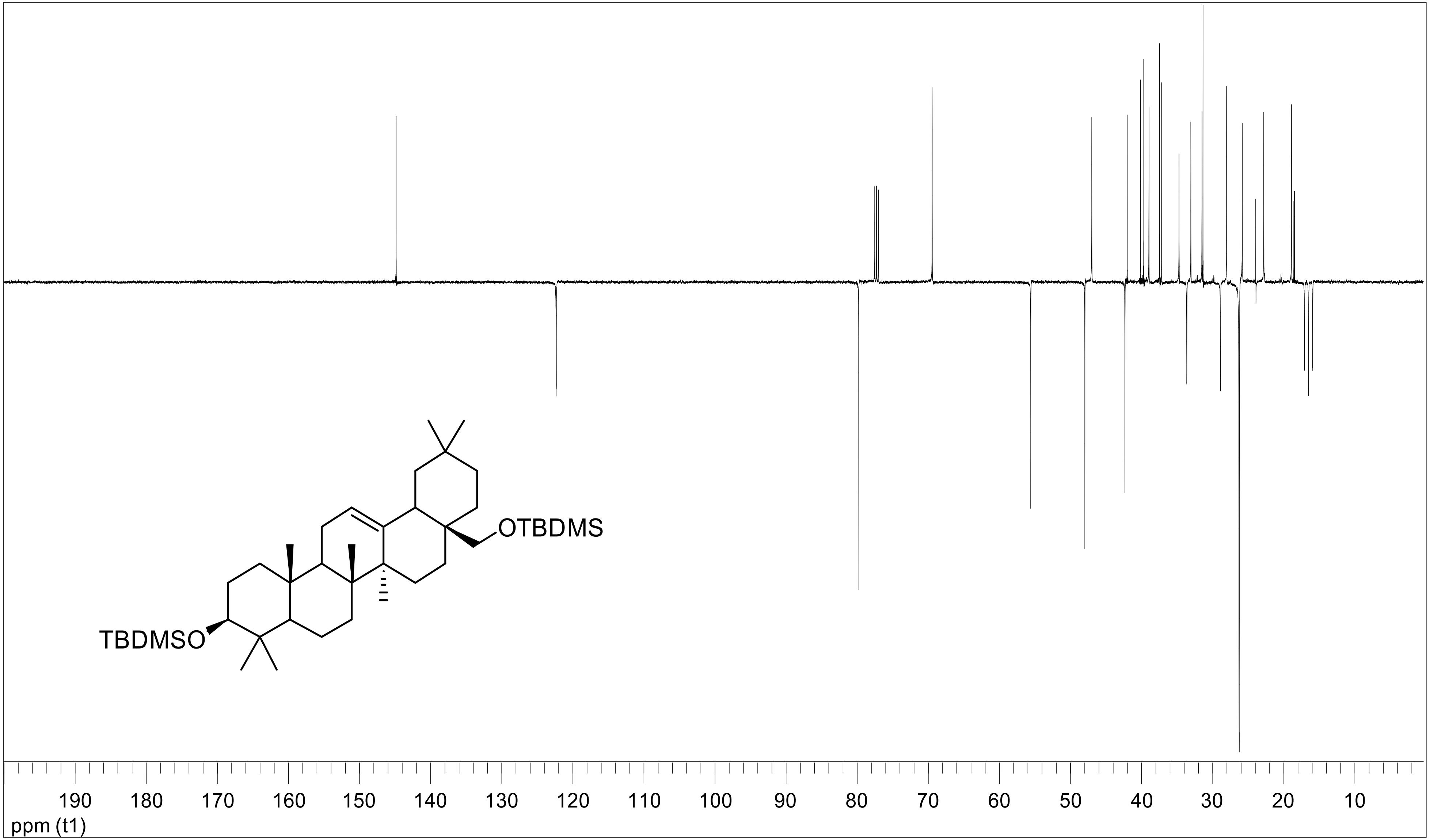

Figure S8: ^{13}C-APT NMR spectrum of compound **3** (125 MHz, $CDCl_3$)

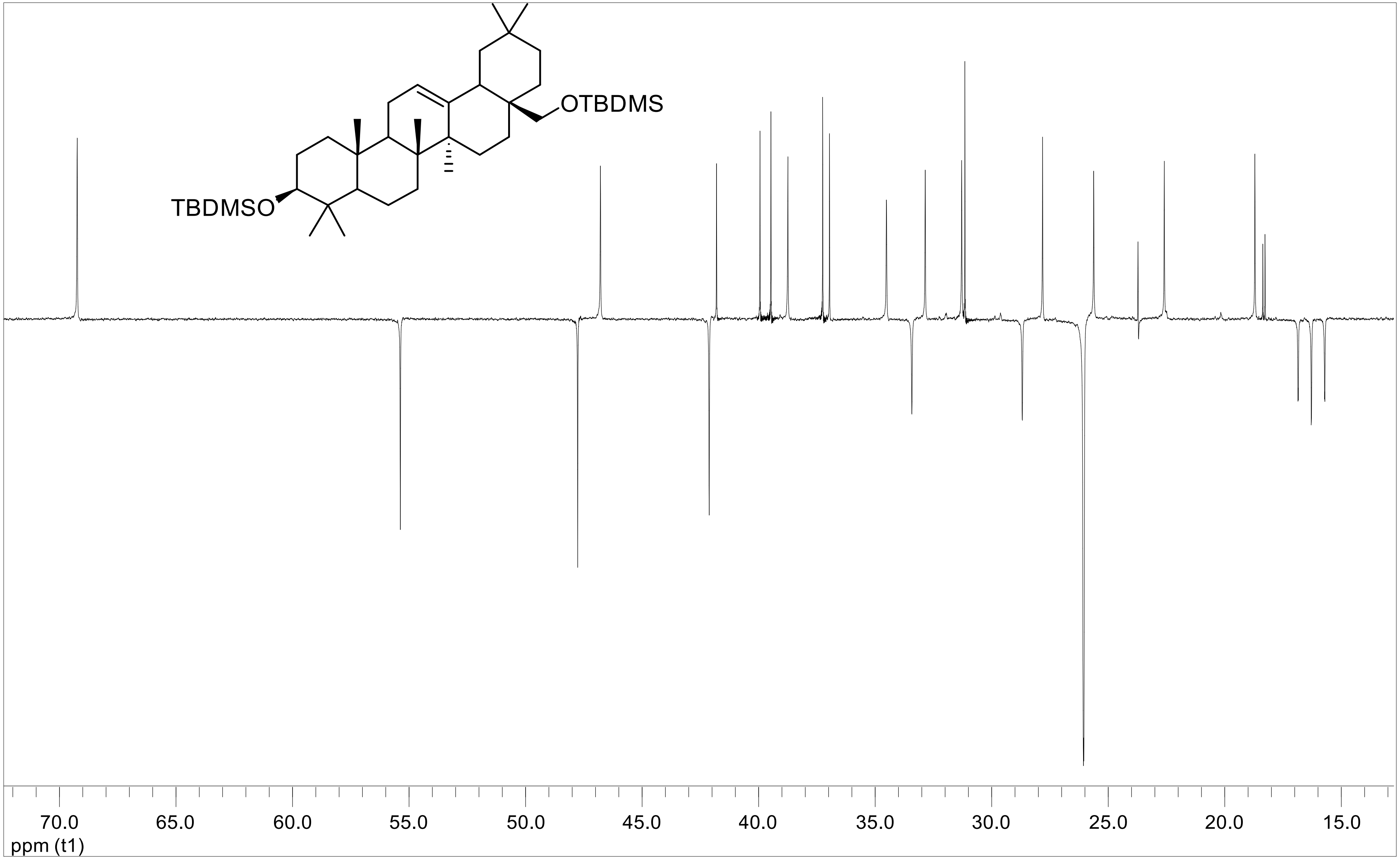

Figure S9: ^{13}C-APT NMR spectrum of compound **3** (extended aliphatic region) (125 MHz, $CDCl_3$)

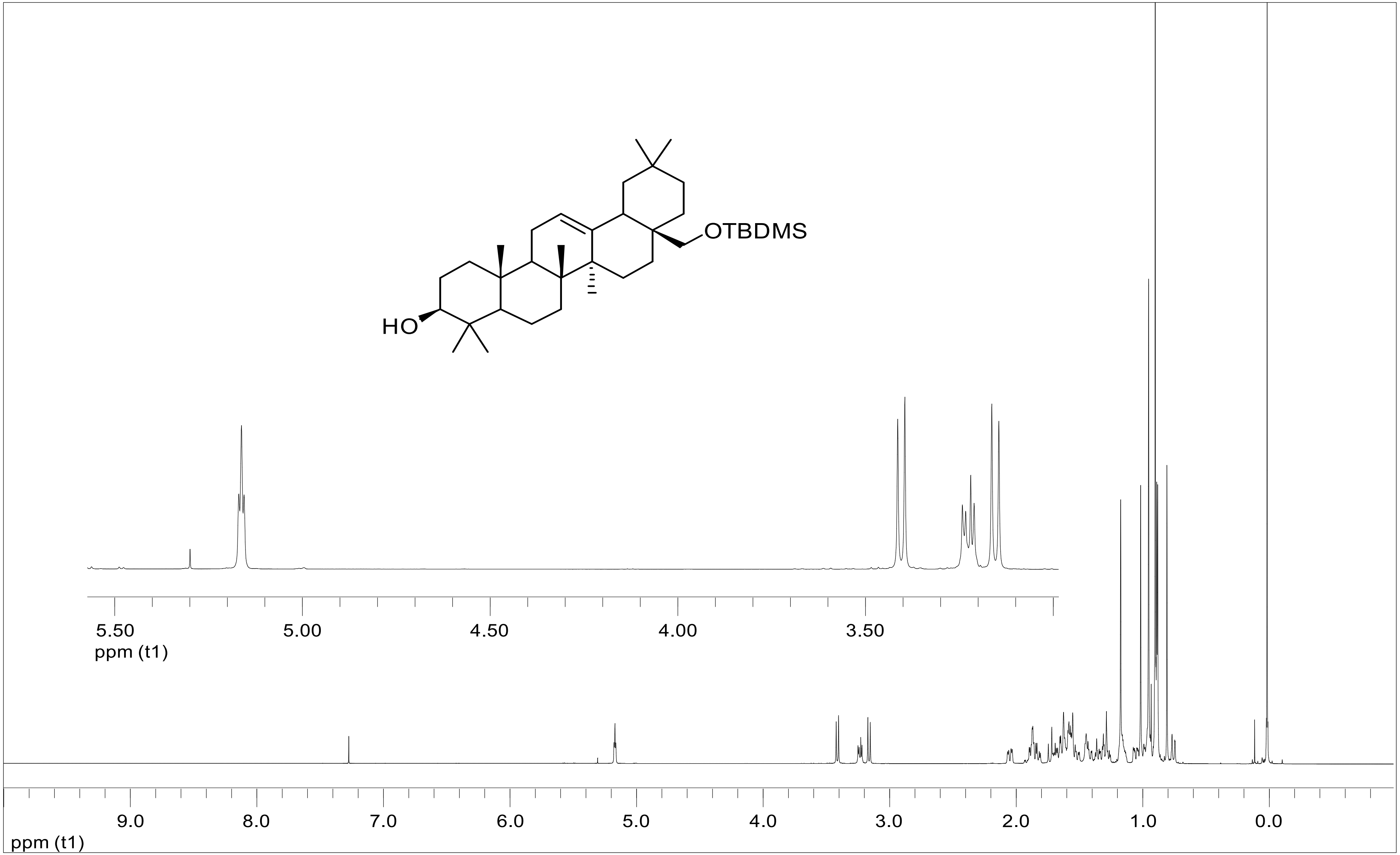

Figure S10: ^{1}H-NMR spectrum of compound **4** (500 MHz, $CDCl_3$)

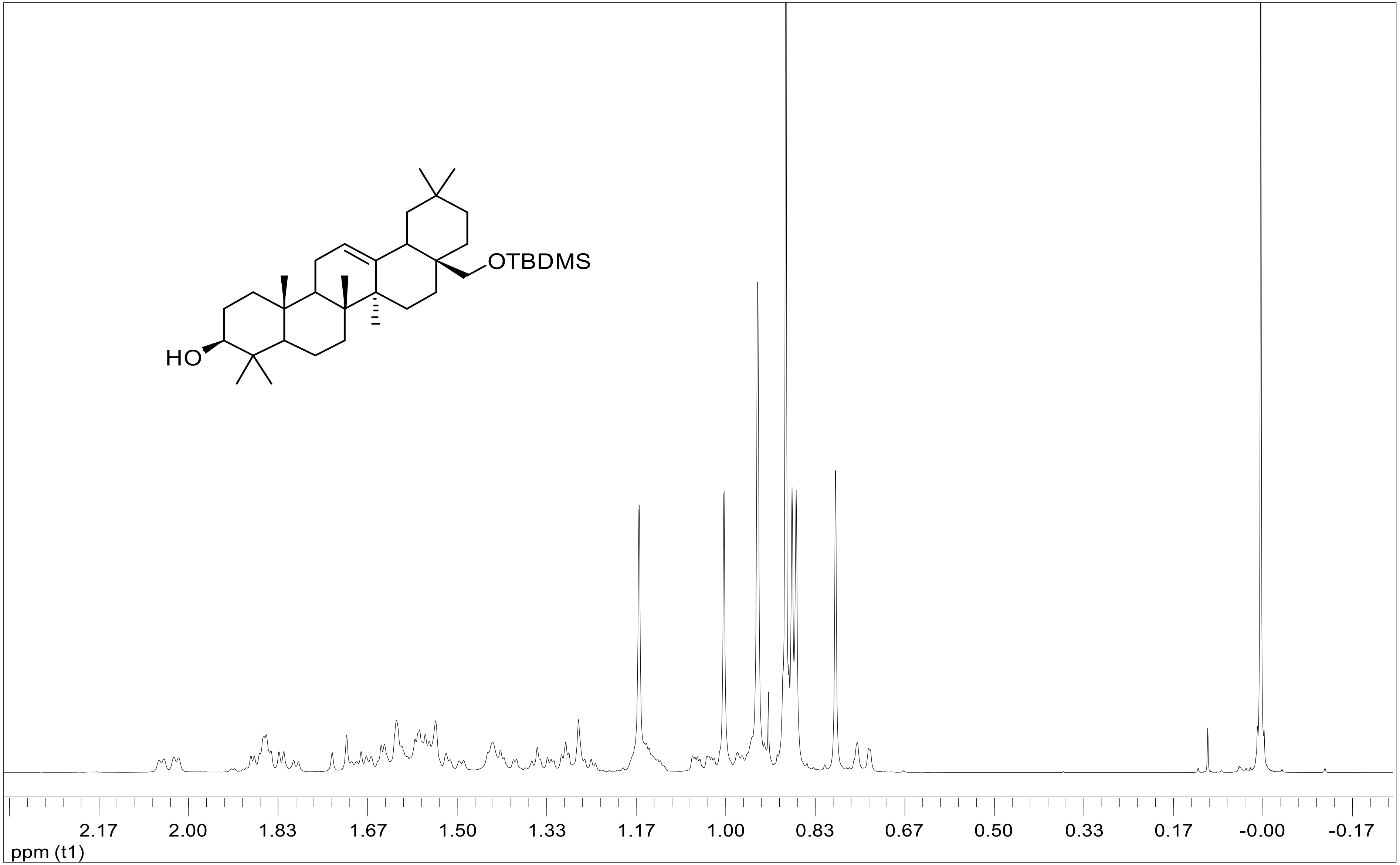

Figure S11: ^{1}H-NMR spectrum of compound **4** (extended aliphatic region) (500 MHz, $CDCl_3$)

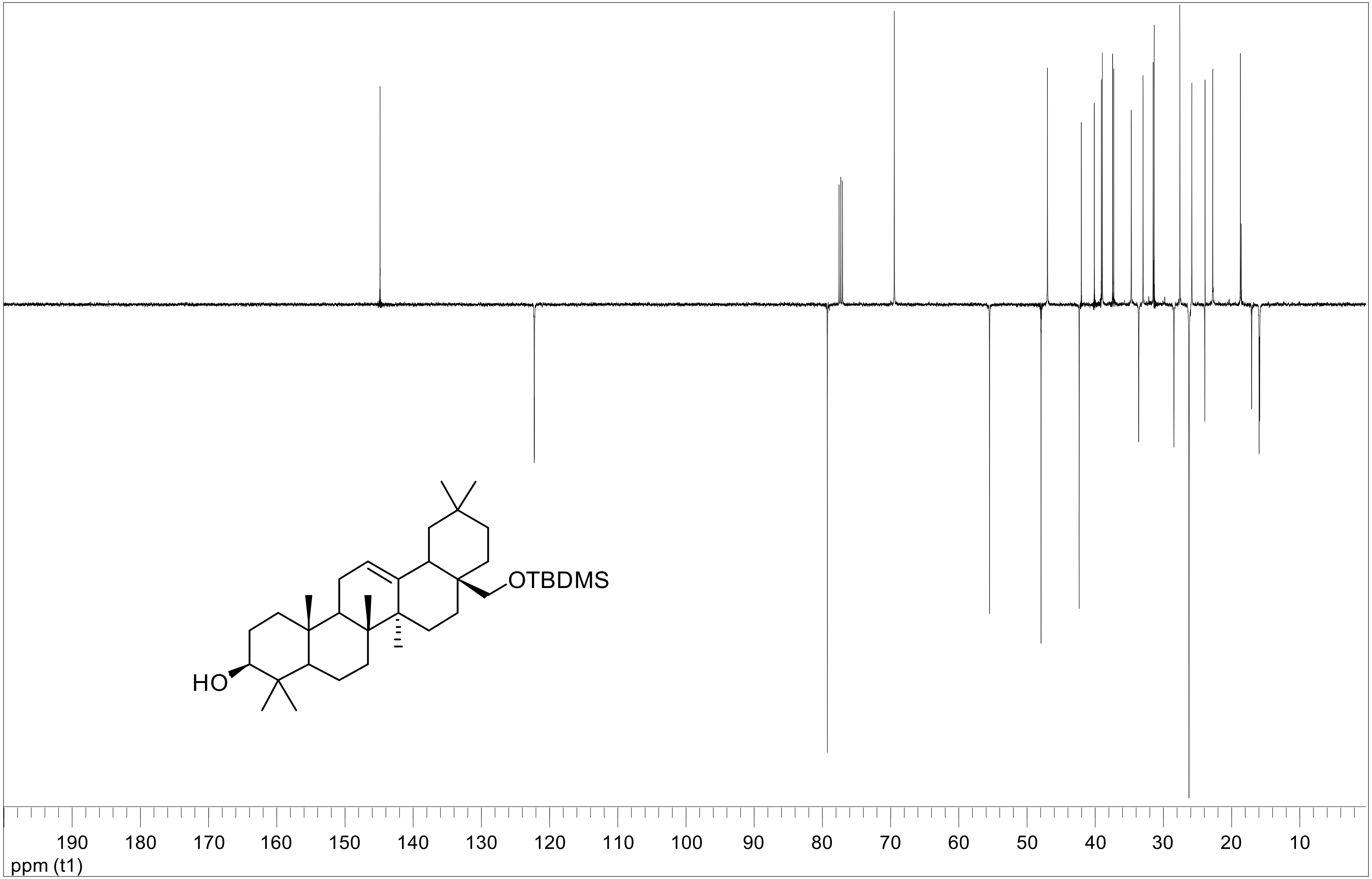

Figure S12: ^{13}C-APT NMR spectrum of compound **4** (125 MHz, $CDCl_3$)

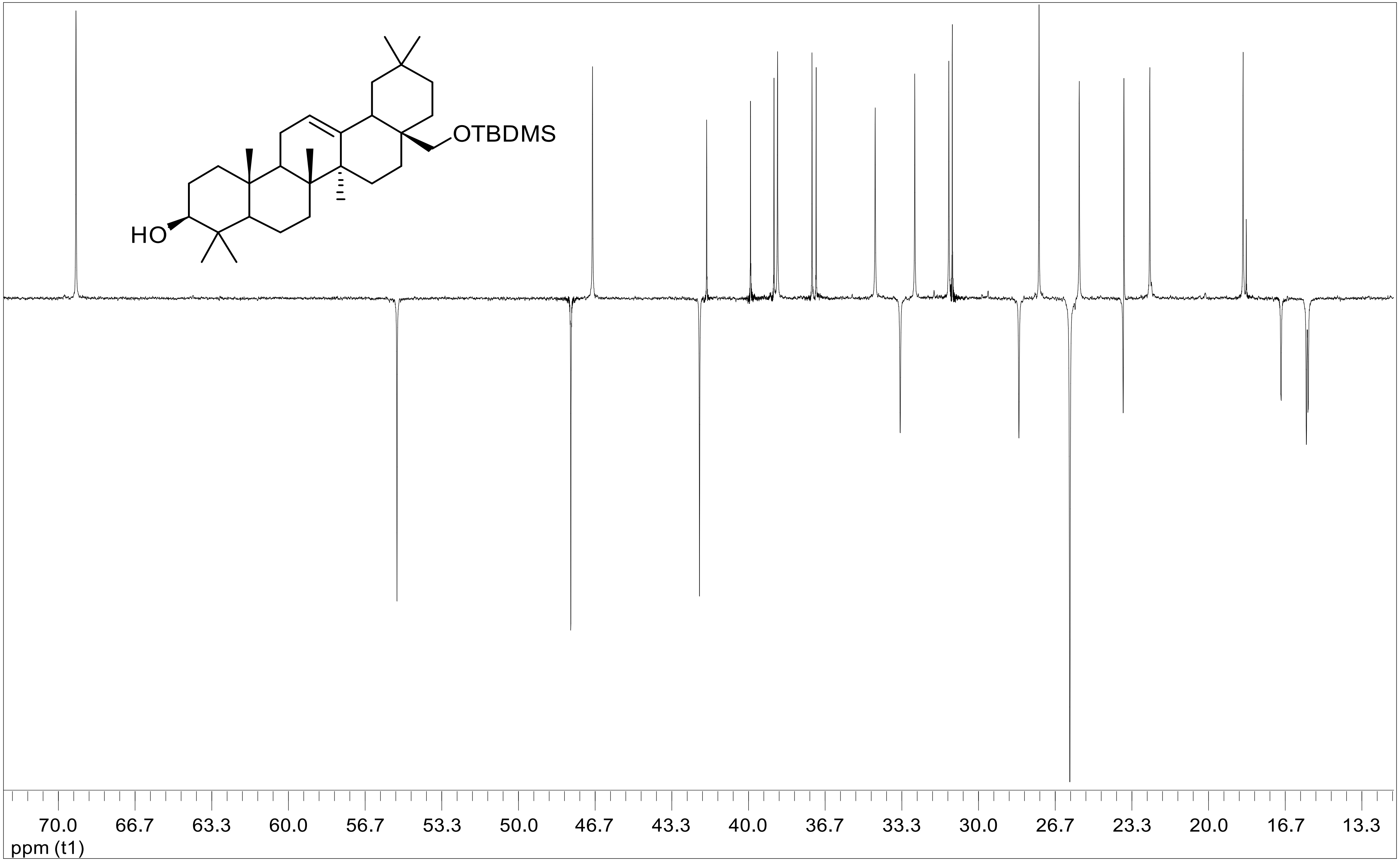

Figure S13: ^{13}C-APT NMR spectrum of compound **4** (extended aliphatic region) (125 MHz, $CDCl_3$)

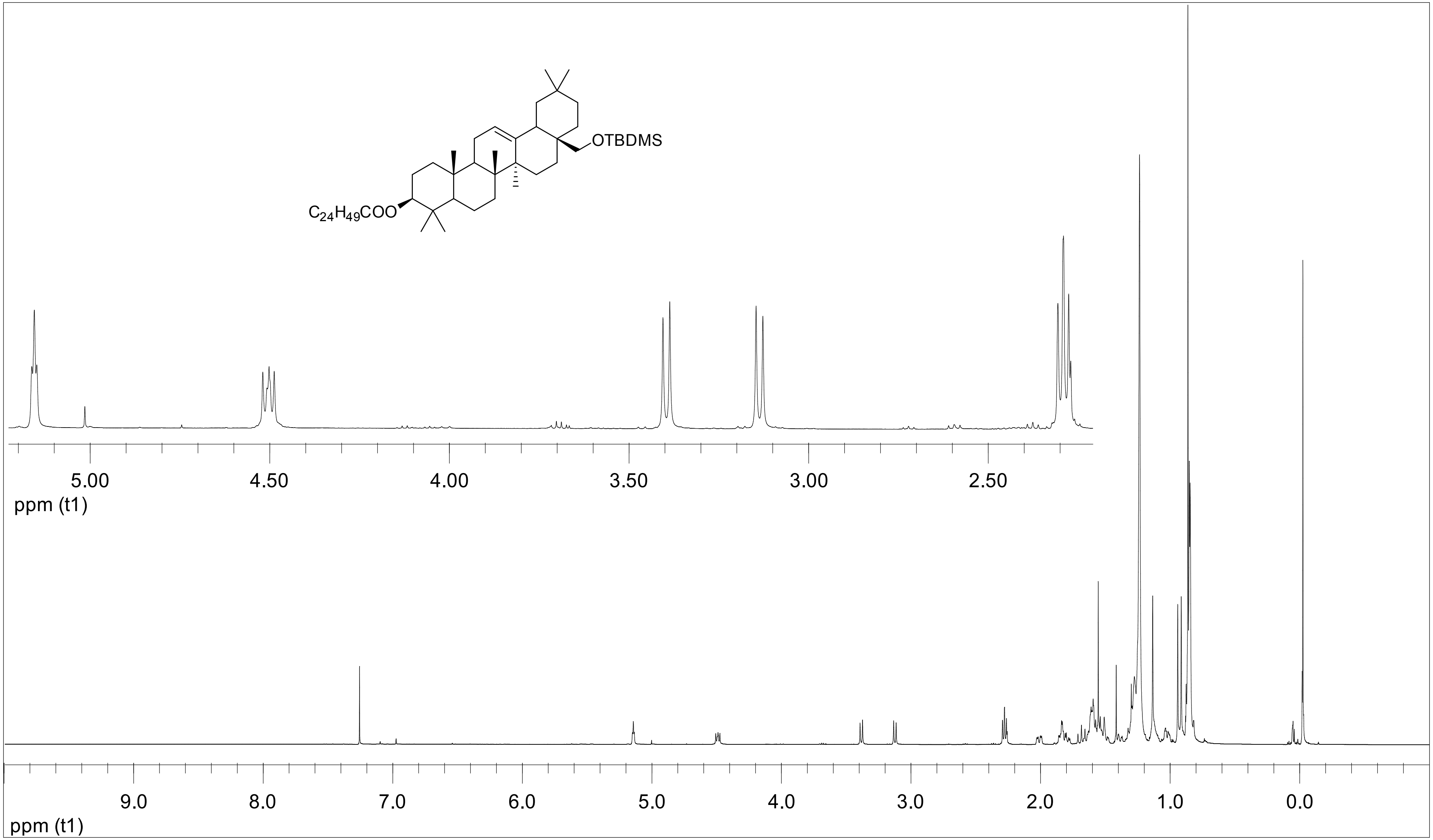

Figure S14: ^{1}H-NMR spectrum of compound **5** (500 MHz, $CDCl_3$)

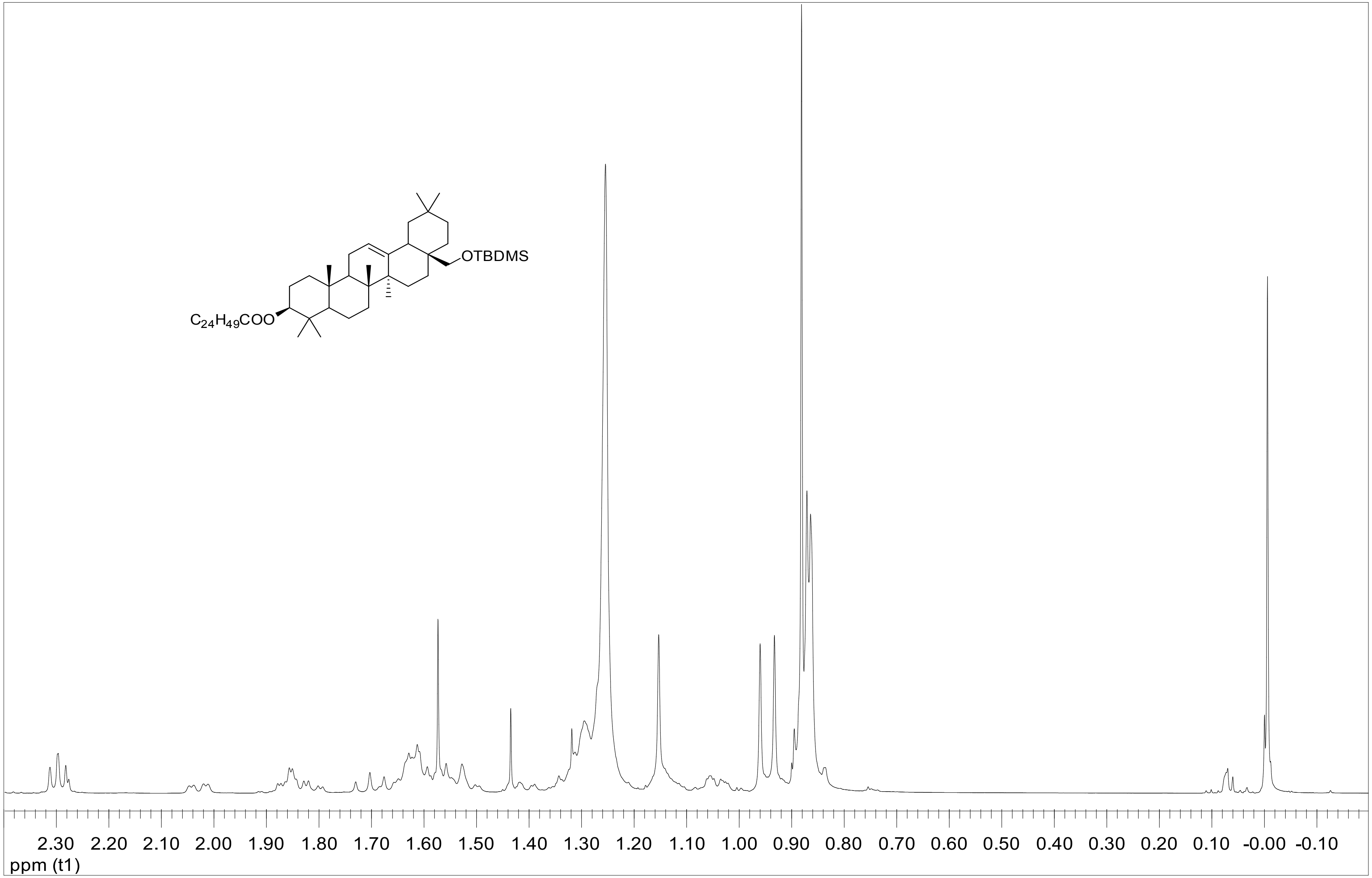

Figure S15: ^{1}H-NMR spectrum of compound **5** (extended aliphatic region) (500 MHz, $CDCl_3$)

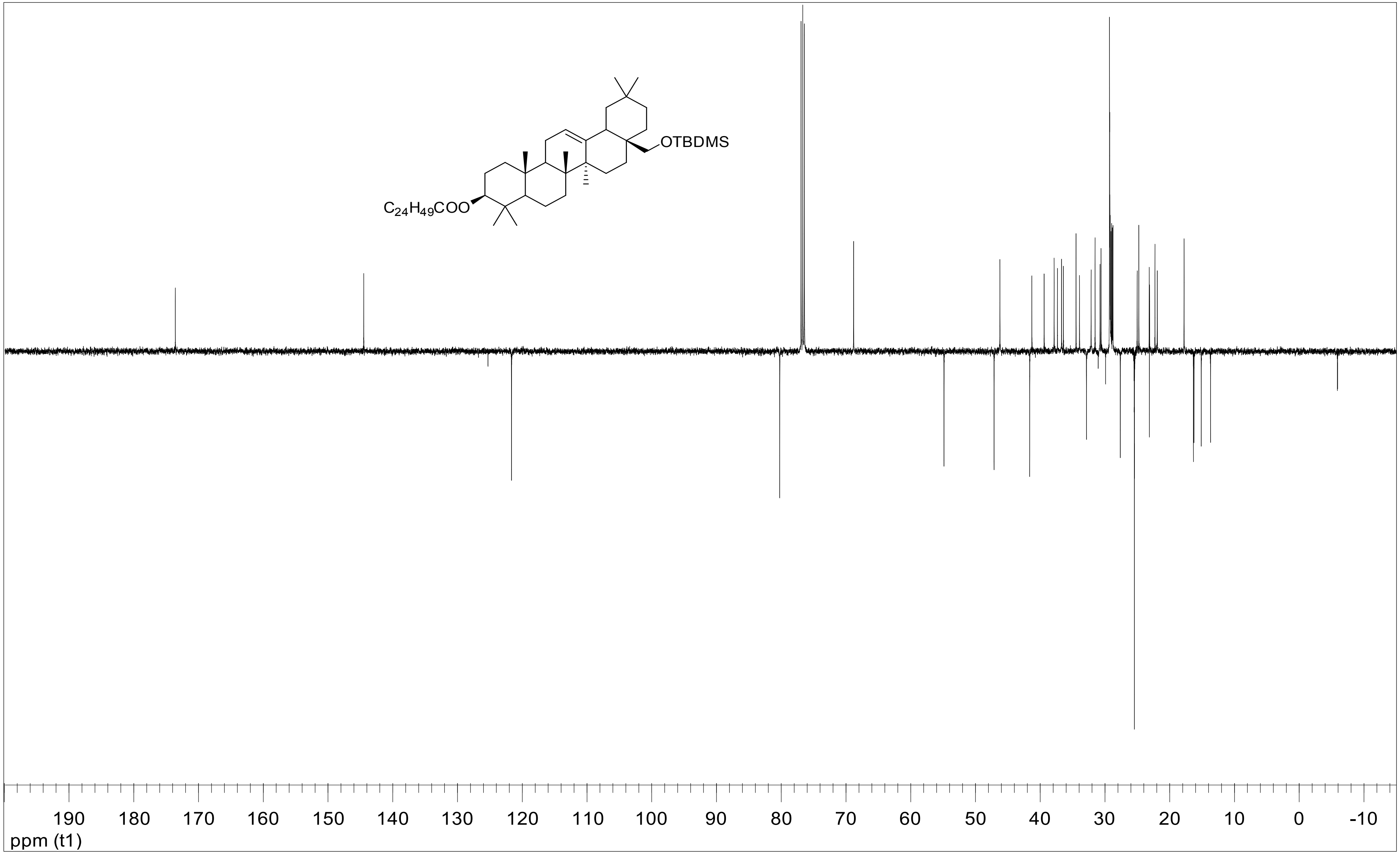

Figure S16: ^{13}C-APT NMR spectrum of compound **5** (125 MHz, $CDCl_3$)

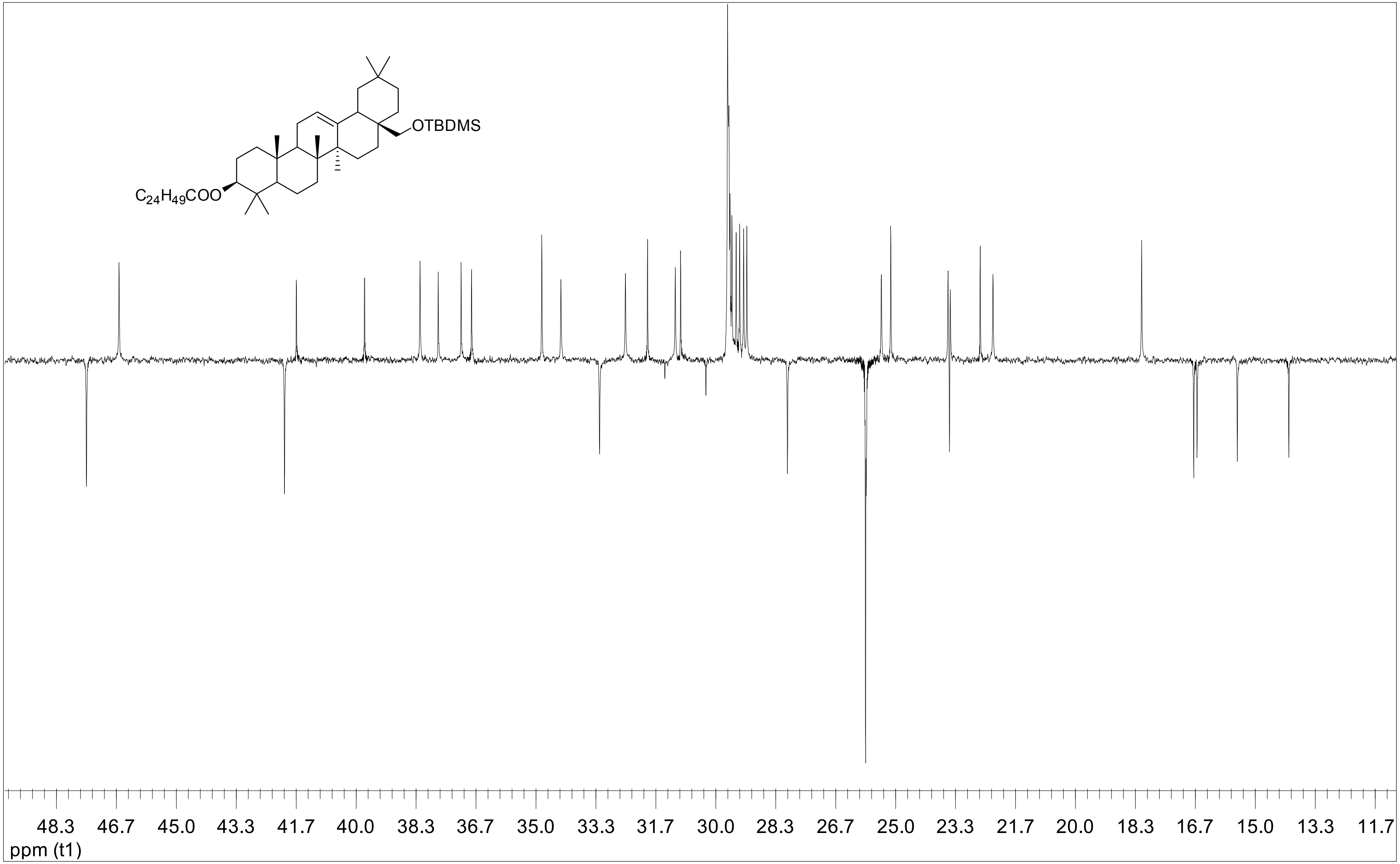

Figure S17: ^{13}C-APT NMR spectrum of compound **5** (extended aliphatic region) (125 MHz, $CDCl_3$)

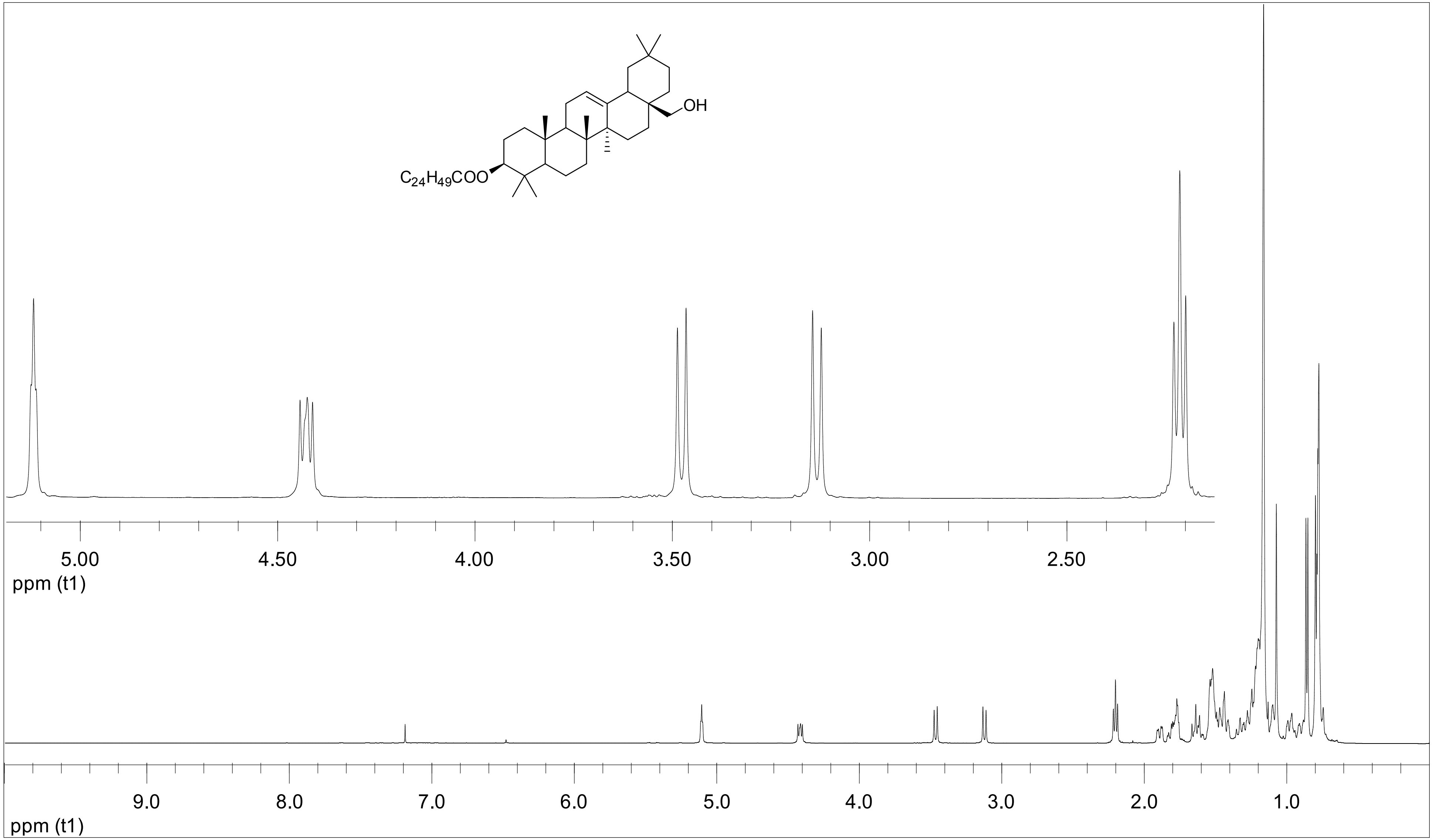

Figure S18: ^{1}H-NMR spectrum of **OPCA** (500 MHz, $CDCl_3$)

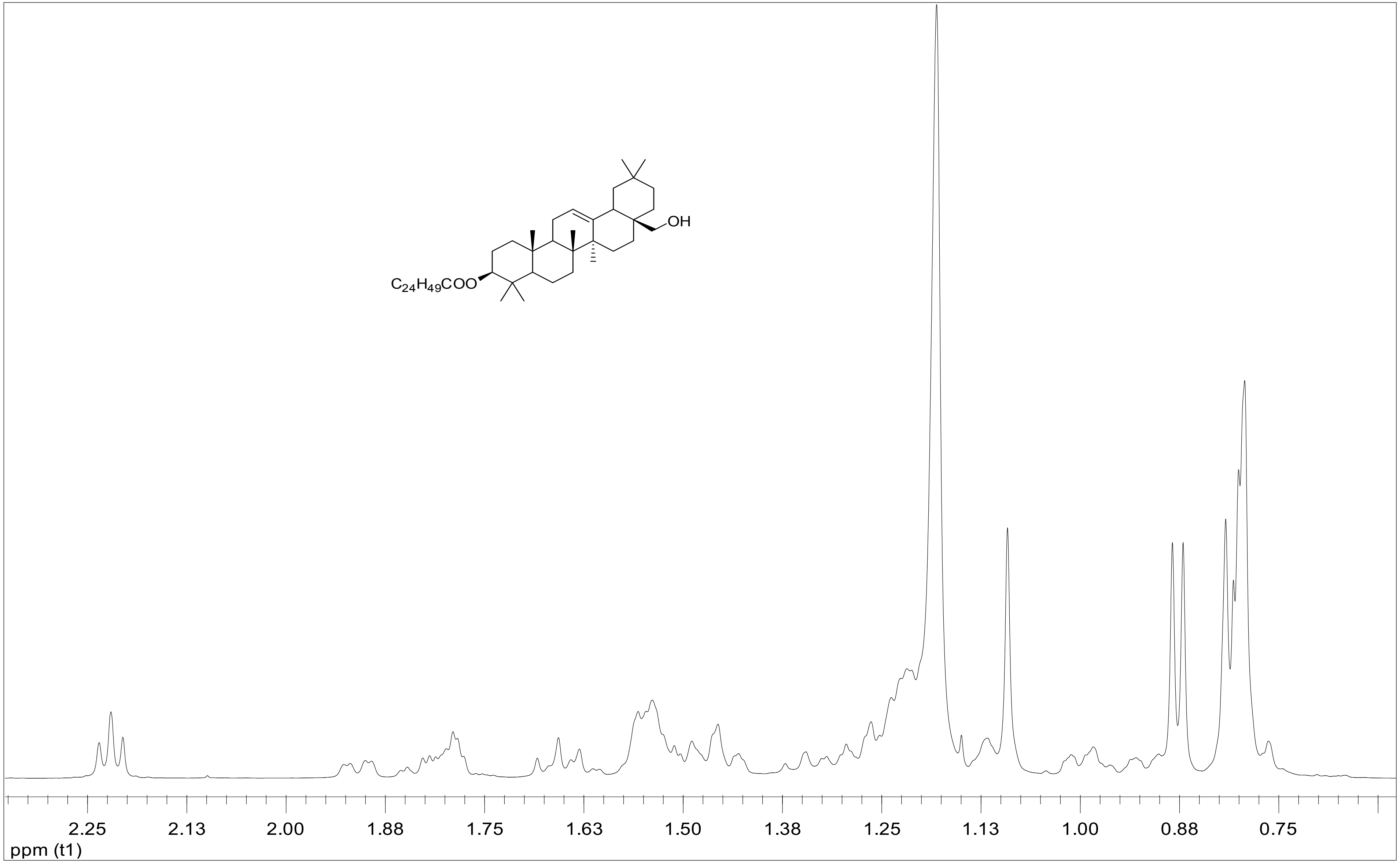

Figure S19: ^{1}H-NMR spectrum of OPCA (extended aliphatic region) (500 MHz, $CDCl_3$)

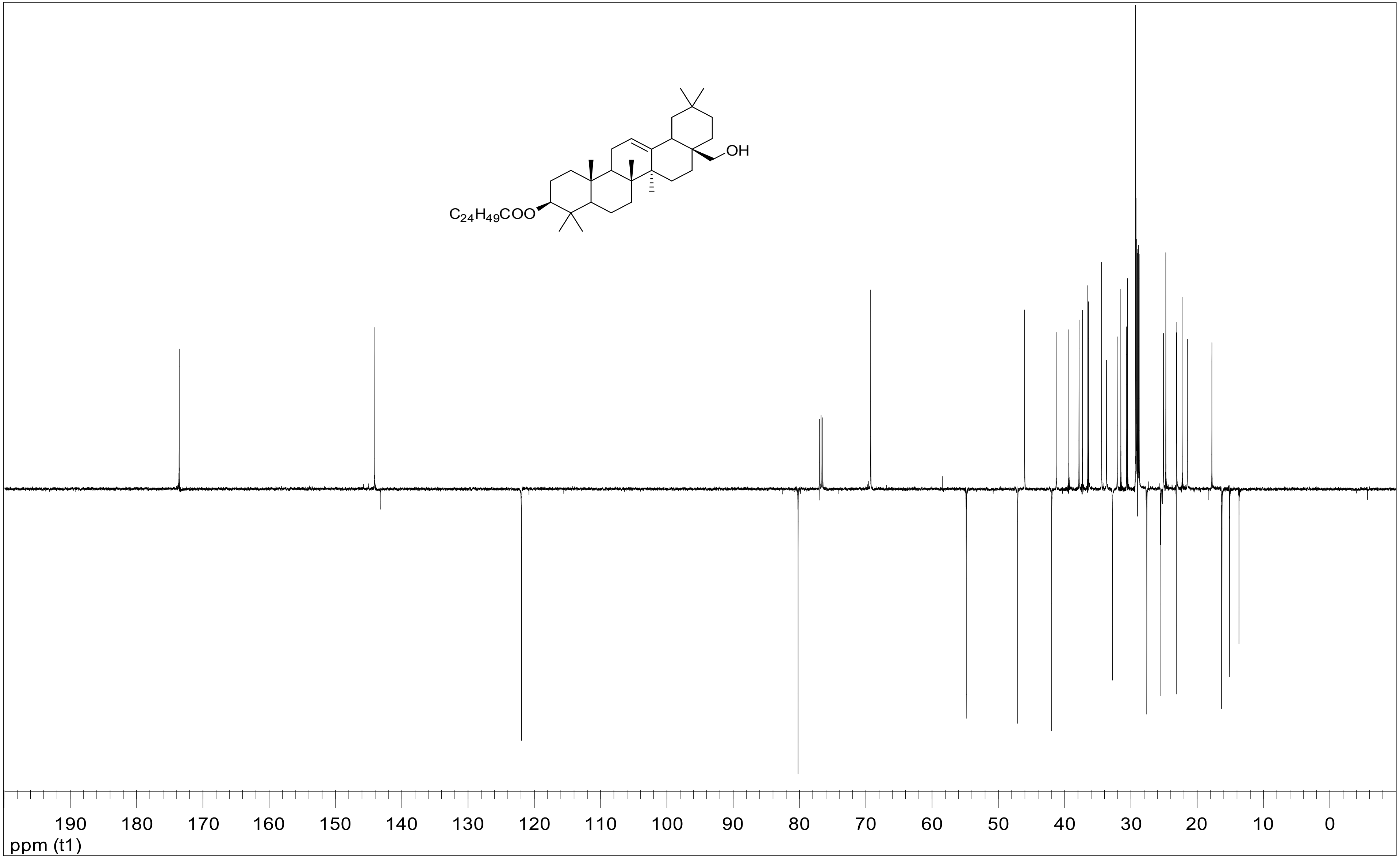

Figure S20: ^{13}C-APT NMR spectrum of **OPCA** (125 MHz, $CDCl_3$)

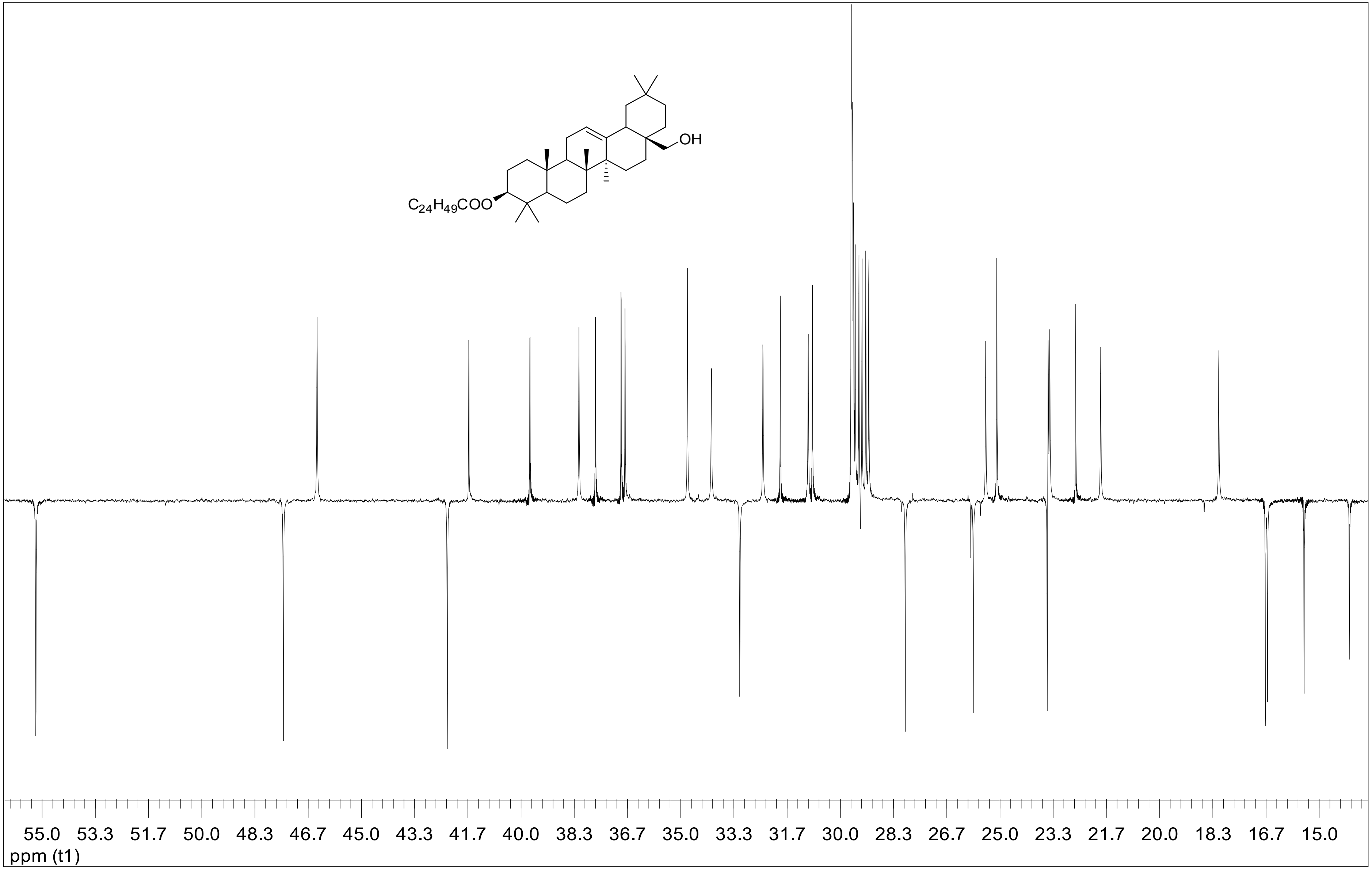

Figure S21: ^{13}C-APT NMR spectrum of **OPCA** (extended aliphatic region) (125 MHz, $CDCl_3$)

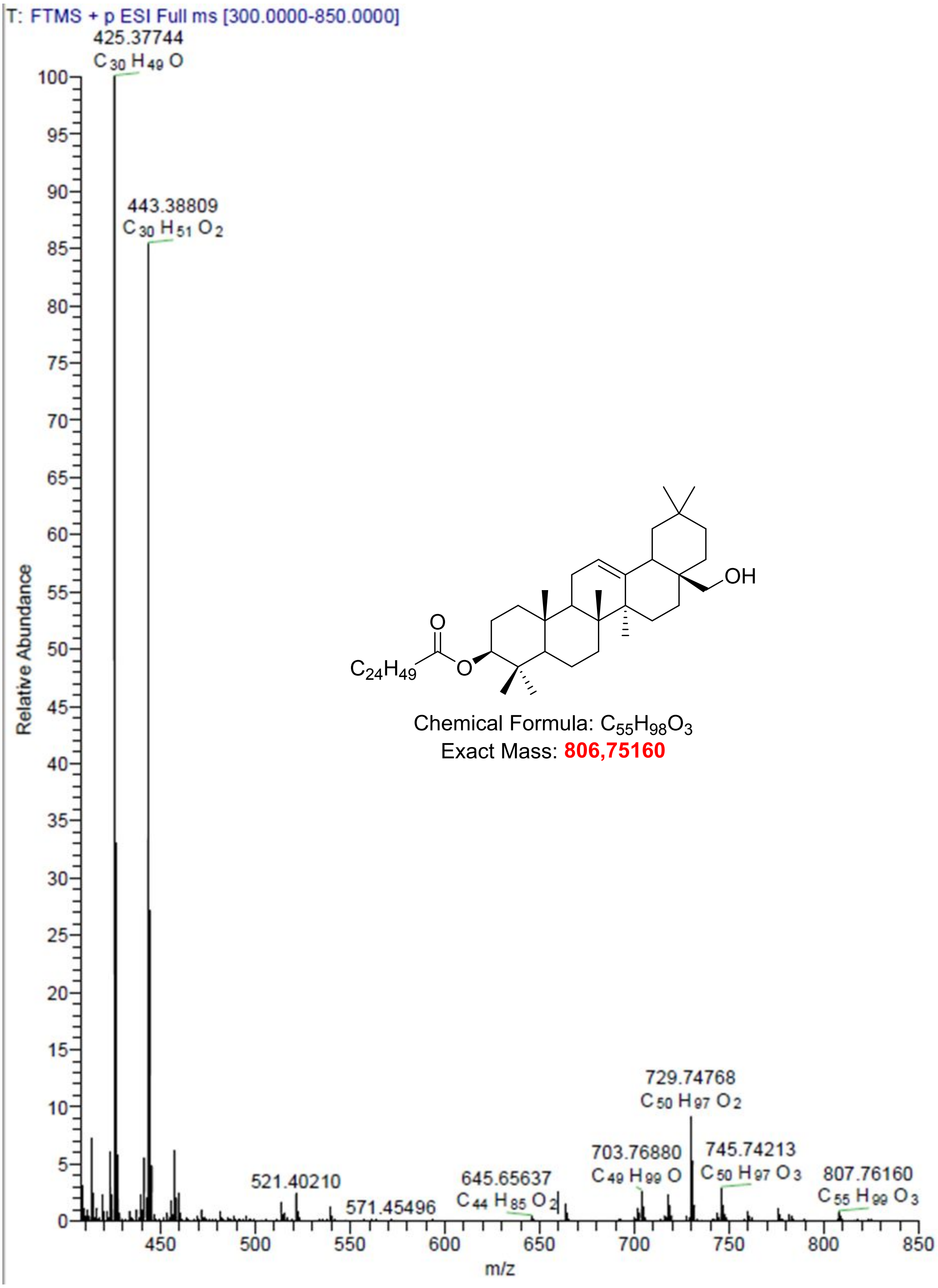

Figure S22: ESI-HRMS Spectrum of **OPCA**

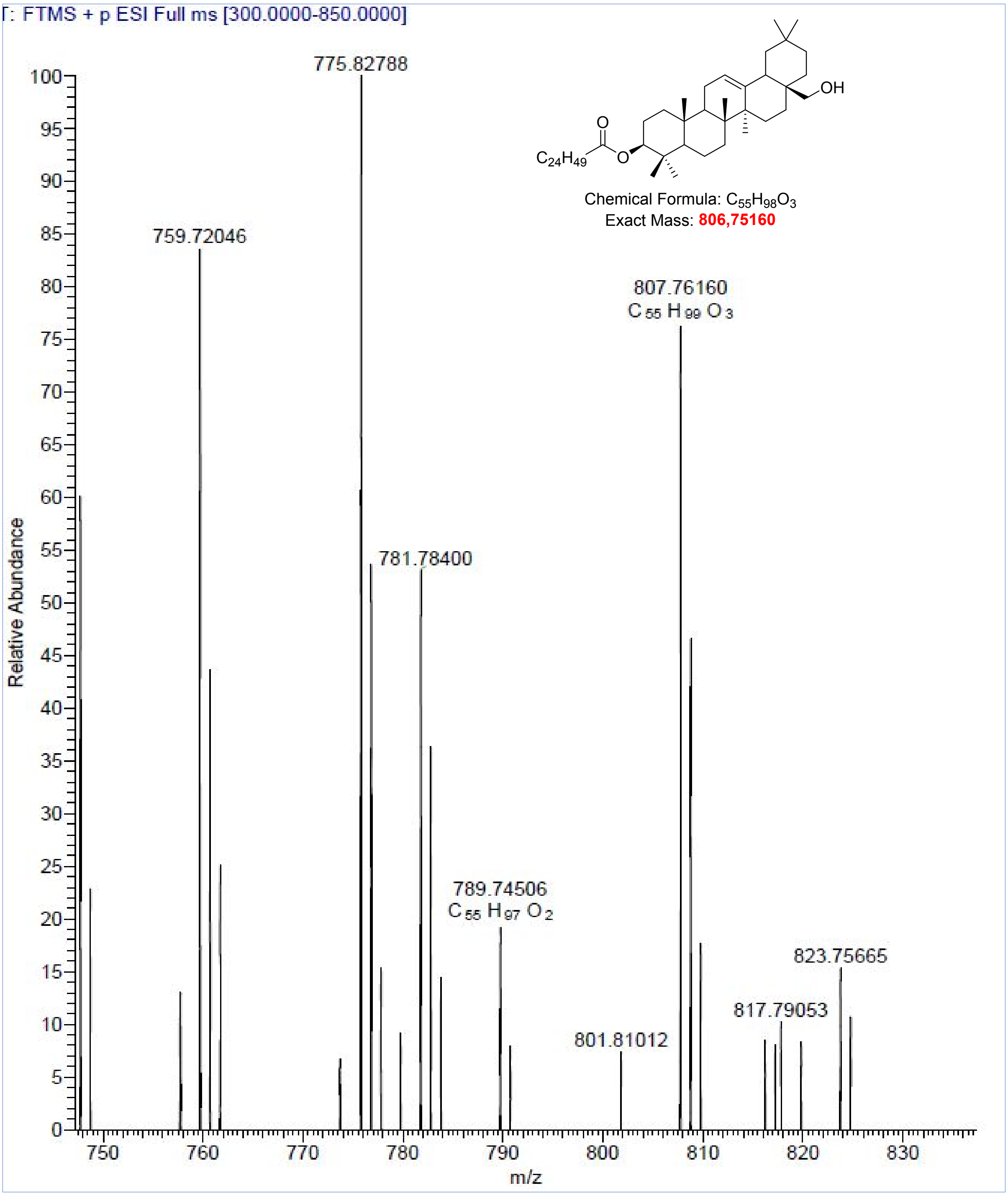

Figure S23: ESI-HRMS Spectrum of **OPCA** (molecular ion peak)

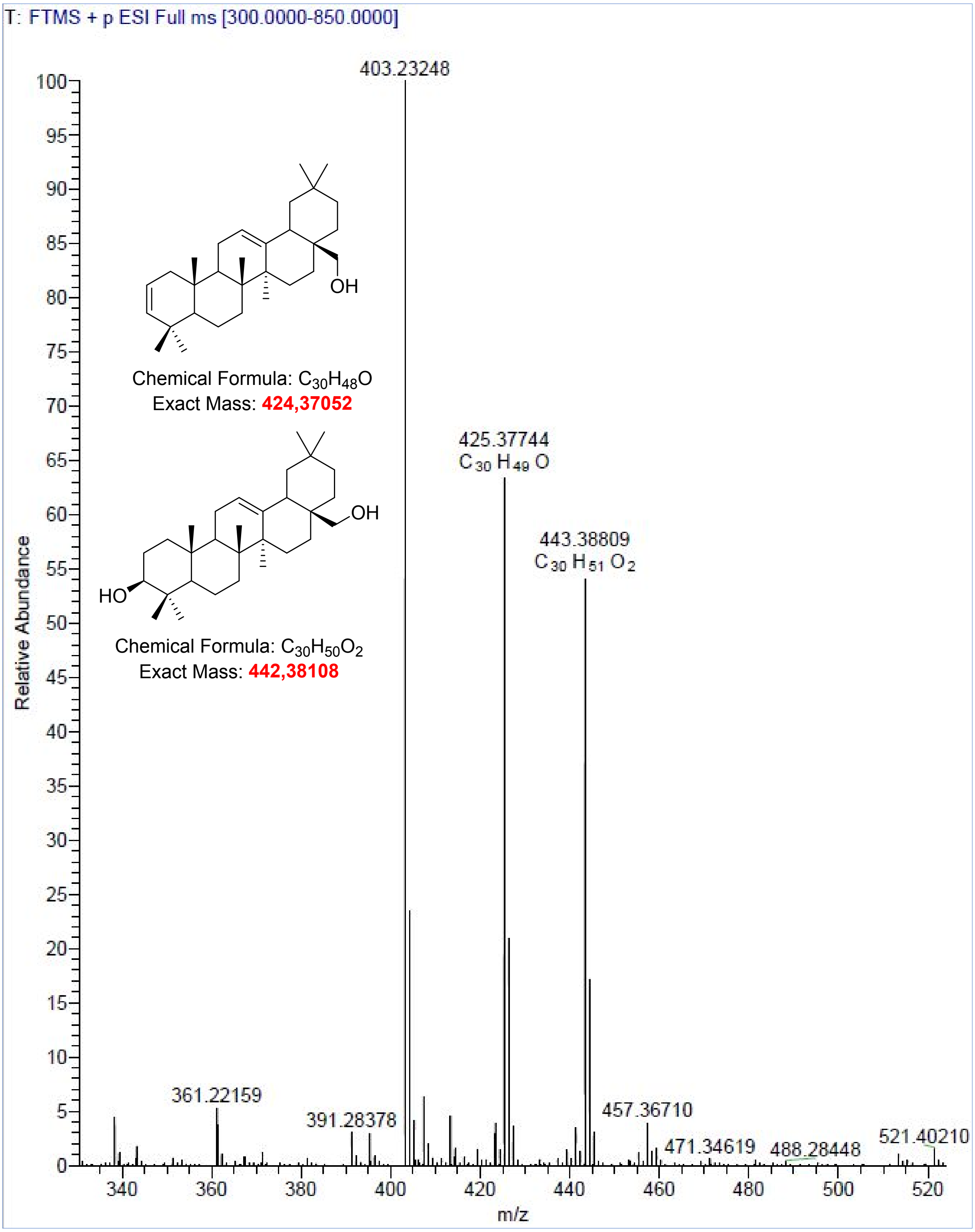

Figure S24: ESI-HRMS Spectrum of **OPCA** (fragments)

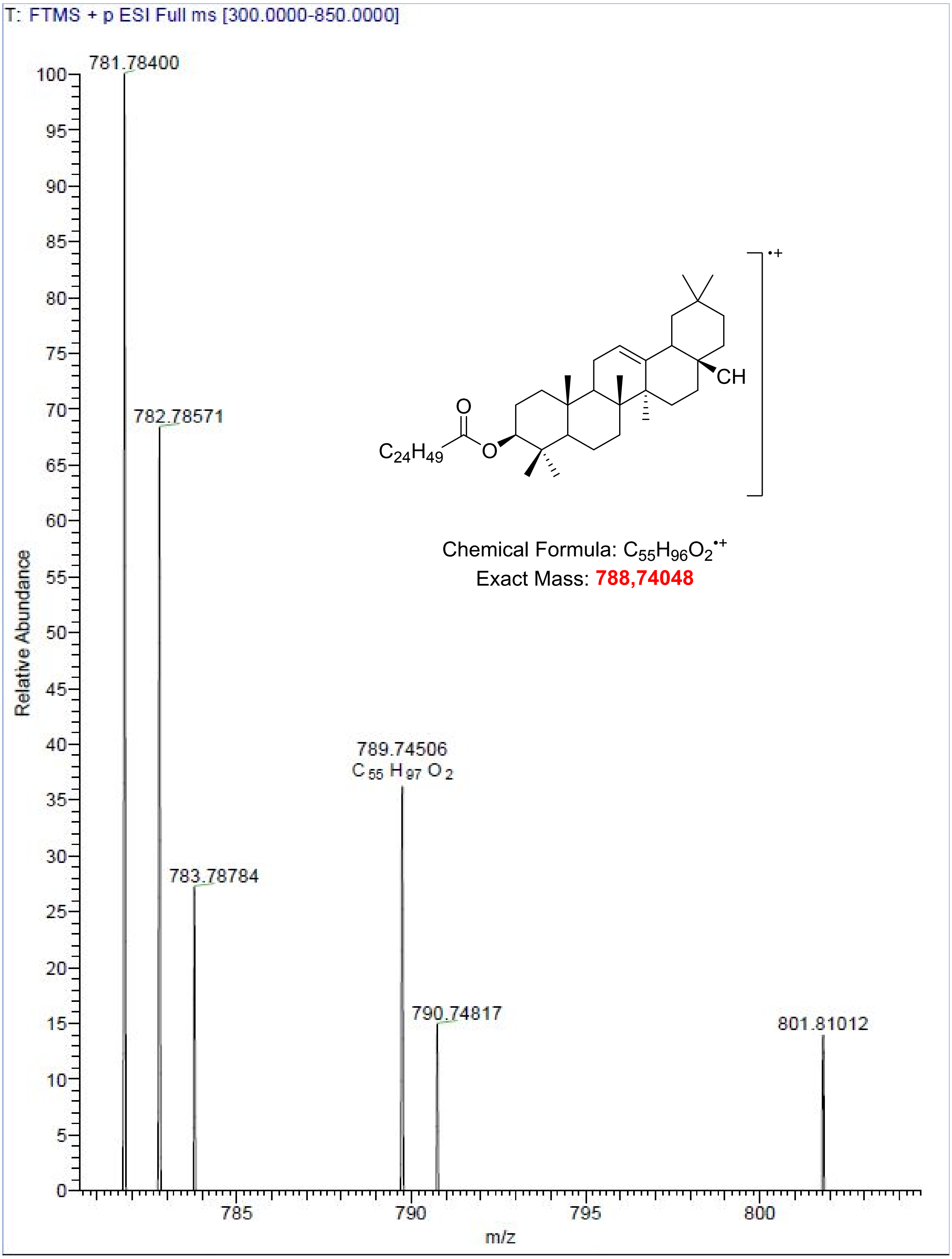

Figure S25: ESI-HRMS Spectrum of **OPCA** (fragments)

Waters

C:\Program Files (x86)\ChromScope IE\LC\Projects\halil şenol\DataFiles\10_21_2022\HSL-ASEN-OPC_10-21-2022 2-15-36 PM.tta

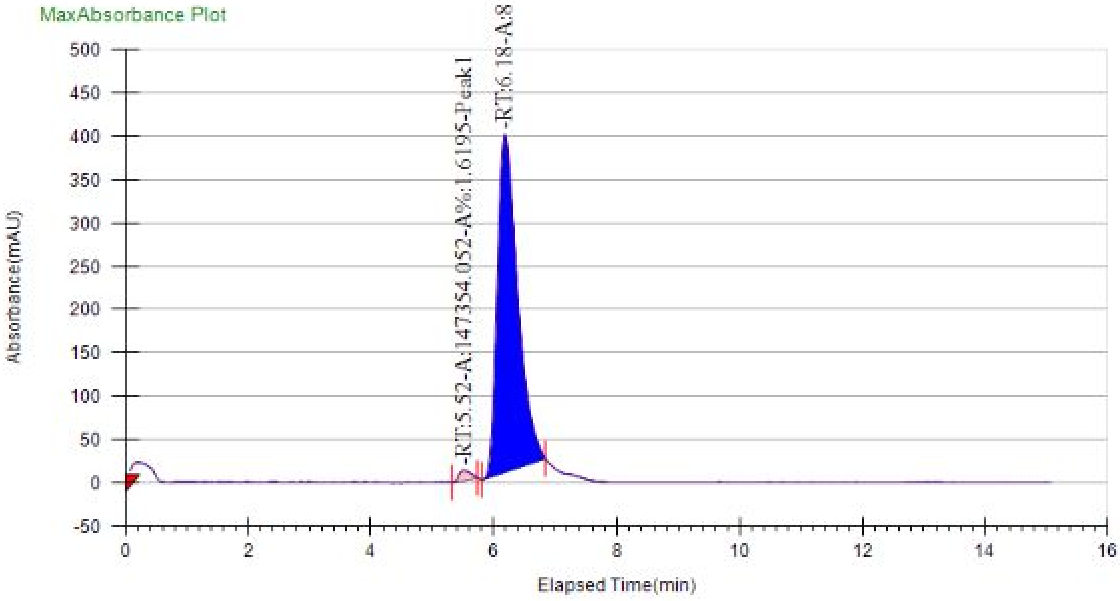

General Information

Log Author	Log Date	Report By	Report Date	Notes
Waters	10/21/2022 2:15:36 PM	Default User	10/21/2022	

Run Information

Instrument Method	Inj. Vol. (uL)	Sample	Well Location	Flow (ml/min)	Pressure
Enjeksiyon-hsl-rbs-8	1500	HSL-ASEN-OPC	1:A,1	3.00	0 Bar

Peak Information

Peak No	% Area	Area	Ret. Time	Height	Cap. Factor
1	1.6195	147354.052	5.52 min	11.5605	0
2	98.3805	8951242.0694	6.18 min	389.5928	0

Waters Corporation

Figure 26. HPLC analysis for the purifty of the synthesized OPCA.

Table S1. Primer sequences for promoter methylation analyses of the inflammatory regulatory genes

**Primer Name**	**Sequence (5'→ 3)**
CX3CL1_FM	TGGTTGGTAGTTTAAAAAGAGTAGC
CX3CL1_FU	TGGTTGGTAGTTTAAAAAGAGTAGTGT
CX3CL1_RM	TAACTAACCCAACTTCTAACTCGAA
CX3CL1_RU	TAACTAACCCAACTTCTAACTCAAA
CXCL12_FM	TCGTATTATTTATTTATTTTTAGTCGA
CXCL12_FU	TTGTATTATTTATTTATTTTTAGTTGA
CXCL12_RM	ATCTCACTACAATCATTCCCGAA
CXCL12_RU	AATCTCACTACAATCATTCCCAAA
CXCL14_FM	AATAGAGTTTAATATCGTAGGGGGC
CXCL14_FU	AGAGTTTAATATTGTAGGGGGTGG
CXCL14_RM	CAAAACACACAAATTTACTATCCGA
CXCL14_RU	AAAACACACAAATTTACTATCCAAA
FADD_FM	TTTTTTTTATATAGTAGGGTTGCGT
FADD_FU	TTTTTTTTATATAGTAGGGTTGTGT
FADD_RM	GAATATAACTATAACCGCTTCCGC
FADD_RU	CAAATATAACTATAACCACTTCCACC
IL10RA_FM	GGAAGATAATTGAGGAAGAAATATCG
IL10RA_FU	AAGATAATTGAGGAAGAAATATTGT
IL10RA_RM	ACGTAACACAAATACCCAAACGTA
IL10RA_RU	AACATAACACAAATACCCAAACATA
IL11_FM	GAGGGGACGTTAATGATTTTATC

IL11_FU	GAGGGGATGTTAATGATTTTATTG
IL11_RM	GACTACCTAACAAAACTCACACCG
IL11_RU	AACTACCTAACAAAACTCACACCAC
IL13_FM	GGATATTGATTTAGCGGTTTAGTC
IL13_FU	GATATTGATTTAGTGGTTTAGTTGG
IL13_RM	ATAAAAACGCGACCTTATATAACGA
IL13_RU	TAAAAACACAACCTTATATAACAAA
IL17RA_FM	TTTTTTTGTAAAACGGTGTATAGTC
IL17RA_FU	TTTTTGTAAAATGGTGTATAGTTGT
IL17RA_RM	ATAAAACACAAAACCCCTTAACGT
IL17RA_RU	ATAAAACACAAAACCCCTTAACATT
IL18_FM	TTATTTTTGGTTTTATTCGTAAGC
IL18_FU	TATTTTTGGTTTTATTTGTAAGTGG
IL18_RM	GCGAAACTATCACGTAACTCG
IL18_RU	CCCACAAAACTATCACATAACTCAC
IL6RA_FM	CGGTTGTAGGAAGTAATTTTTTTC
IL6RA_FU	TGGTTGTAGGAAGTAATTTTTTTTG
IL6RA_RM	ACTCAAATCGATATAAACGCGAC
IL6RA_RU	TAACTCAAATCAATATAAACACAAC
IL6ST_FM	GGGAAGATAGTTTAGGGAGATAGTC
IL6ST_FU	GGGAAGATAGTTTAGGGAGATAGTTG
IL6ST_RM	CACTTAAATAAATAACCGAAACGAA
IL6ST_RU	CACTTAAATAAATAACCAAAACAAA
SOCS5_FM	AGGTTAAGAAGCGGTGGTAATTAC
SOCS5_FU	GGTTAAGAAGTGGTGGTAATTATGG

SOCS5_RM	TACCACGAAAATAAATCATACACGA
SOCS5_RU	ACCACAAAAATAAATCATACACAAA
TGFB1_FM	GGTGGTGGGTTATAGTTGTTGTAC
TGFB1_FU	GGTGGTGGGTTATAGTTGTTGTAT
TGFB1_RM	CTAACAATCTCCAAAATCCACGTA
TGFB1_RU	AACTAACAATCTCCAAAATCCACATA

FU, forward primer unmethylated promoter; RU, reverse primer unmethylated promoter; FM, forward primer methylated promoter; RM, reverse primer methylated promoter.
